# Supplementary material for: Particle-like topologies in light
Source: Nat Commun. 2021 Nov 22;12:6785. doi: 10.1038/s41467-021-26171-5 (PMC8608860; doi:10.1038/s41467-021-26171-5)
Supplement: Supplementary file 1 — Supplementary Information [file 41467_2021_26171_MOESM1_ESM.pdf]

Supplementary Information: Particle-like topologies in light

Danica Sugic,[1,2,3] Ramon Droop,[4] Daniel Ehrmanntraut,[4] Eileen Otte,[4]
Franco Nori,[3,5] Janne Ruostekoski,[6] Cornelia Denz,[4] and Mark R Dennis[1,2,7]

[1]*School of Physics and Astronomy, University of Birmingham, Birmingham B15 1TT, UK*
[2]*H H Wills Physics Laboratory, University of Bristol, Bristol BS8 1TL, UK*
[3]*Theoretical Quantum Physics Laboratory, RIKEN Cluster for Pioneering Research, Wako-shi, Saitama 351-0198, Japan*
[4]*Institute of Applied Physics and Center for Nonlinear Science (CeNoS), University of Münster, 48149 Münster, Germany*
[5]*Physics Department, University of Michigan, Ann Arbor, Michigan 48109-1040, USA*
[6]*Physics Department, Lancaster University, Lancaster LA1 4YB, UK*
[7]*EPSRC Centre for Doctoral Training in Topological Design, University of Birmingham, Birmingham B15 2TT, UK*

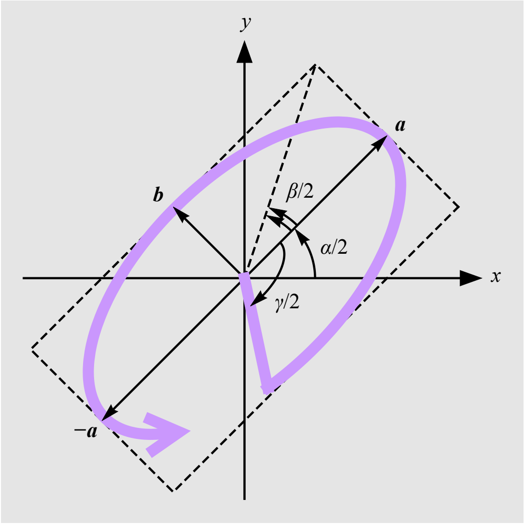

Supplementary Fig. 1. **Polarization ellipse.** A typical polarization ellipse, bounded by a rectangle. The ellipse has a major axis given by vector $\boldsymbol{a}$ and a minor axis vector $\boldsymbol{b}$; their ordering is consistent with the right-handed ellipse orientation (i.e. by $\mathrm{sign}(\boldsymbol{a} \times \boldsymbol{b}) \cdot \hat{\boldsymbol{z}}$). $\boldsymbol{a}$ is chosen rather than its opposite $-\boldsymbol{a}$. The major axis of the ellipse therefore has length a and the minor axis has length b, so the bounding rectangle has side lengths $2a$ and $2b$. The normalization $1 = |E_\mathrm{R}|^2 + |E_\mathrm{L}|^2 = a^2 + b^2$ implies that the bounding box for all ellipses has a fixed diagonal length of 2. The orientation of the major axis of the ellipse (and hence the longer edge of the rectangle) is $\alpha/2$, defined over a range $-\pi/2 < \alpha/2 \leq \pi/2$. Signed by ellipse handedness, half the area of the bounding rectangle is $\pm 2ab = \cos\beta = S_3$. The phase angle on the ellipse is determined by $\gamma/2$; geometrically, this corresponds to the ellipse auxiliary angle. To determine the full range of phase, $-\pi < \gamma/2 \leq \pi$, it is necessary to pick a reference major axis direction $\pm\boldsymbol{a}$ at which $\gamma = 0$.

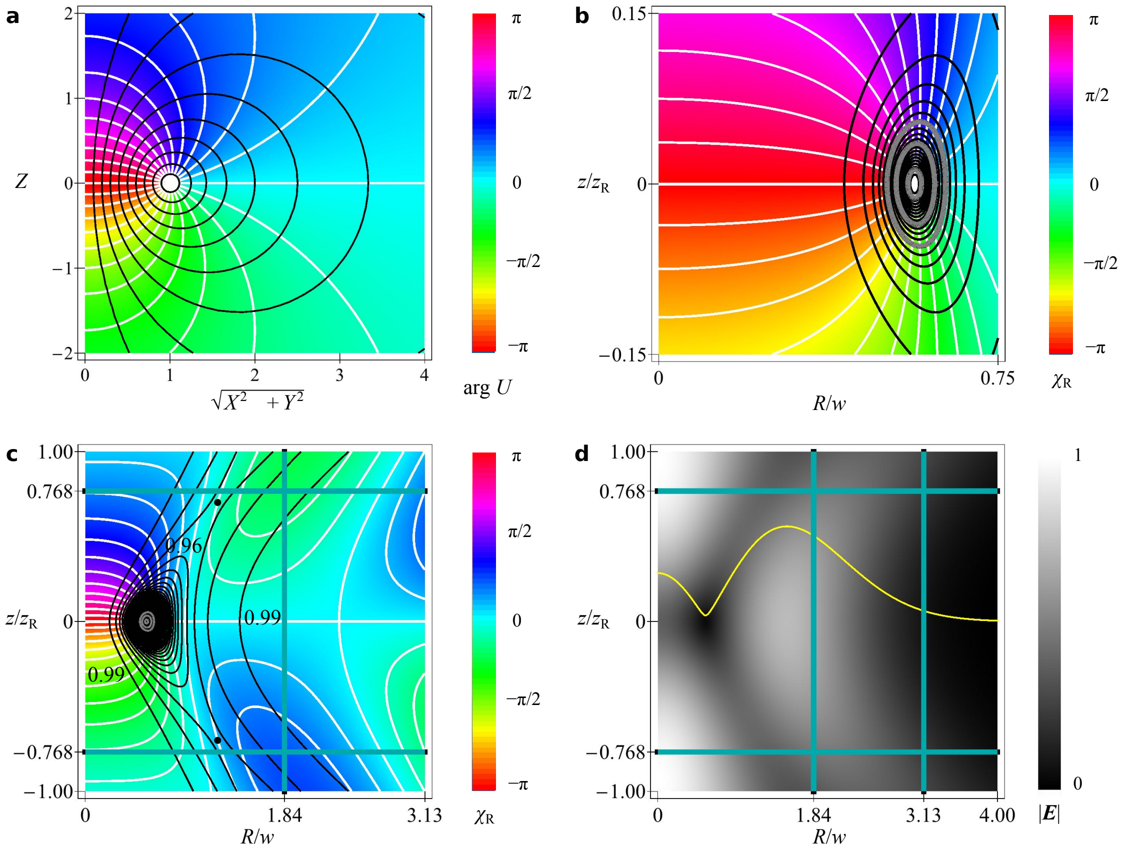

Supplementary Fig. 2. **Hopfion-like field sections of constant azimuth.** **a**, Section of constant φ of the complex field $U(X,Y,Z)$, representing the 3-sphere stereographically projected to infinite euclidean 3-space. The cylindrical radius $\sqrt{X^2+Y^2}$ is on the horizontal axis, and Z is vertical. The black contours denote contours of $|U|$, and the white lines (and colors) contours of phase $\arg U$. The phase is singular on the nodal line $U=0$, on which $X^2+Y^2=1$ and $Z=0$. This is the reference circle of toroidal coordinates φ, σ, τ, and in fact $\sigma = \arg U$, $\cosh\tau = 1/|U|$, so white and black lines represent toroidal coordinates in this plane. **b**, Section of constant ϕ of the model optical field in real space defined in the Methods. The LH C line crosses the plane at its radius $R_0 = 0.57w$. White contours and colors correspond to the axisymmetric phase χ_{R} ($\arg E_{\mathrm{R}}$), and black contours correspond to the axisymmetric values of $S_3 = \cos\beta$. The values of $S_3 = -0.775, 0, 0.398$ chosen in the main text are shown in grey, otherwise the contours of S_3 are at $-1, -0.9, \ldots 0.9, 1$, increasing from the minimum at the C point. The field strongly resembles the Hopf fibration/toroidal configuration of **a**. **c**, Model optical field with larger R and z range plotted. The contours of S_3 are $-1, -0.99, \ldots, 0.99, 1$. The investigated range is indicated by the dark cyan lines showing that the skyrmionic hopfion is mainly inside this range. The Hopf fibration structure breaks down at the saddle points of S_3 which are shown as black dots. **d**, Amplitude of the model optical field across the measured range (see Methods). The yellow line shows the real amplitude profile at the focal plane ($z=0$).

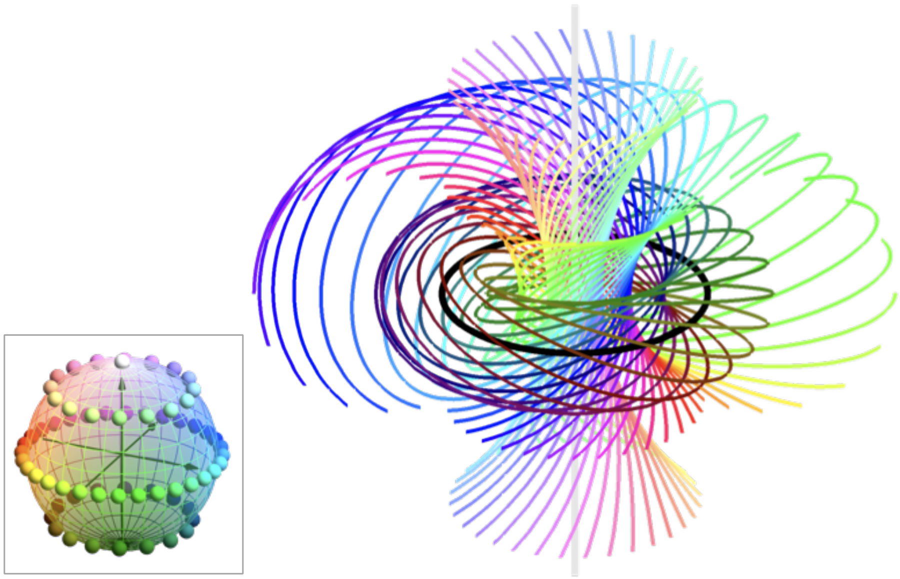

Supplementary Fig. 3. **Hopf fibration.** The 3-sphere, stereographically projected to 3D euclidean space, is filled by circles. The circles are arranged on tori of constant β (equivalently constant S_3, constant τ) which are the toroidal surfaces in toroidal coordinates. Each torus corresponds to a fixed height on the fibration's base space – the Poincaré sphere (inset), with the reference circle (black) corresponding the the south pole (LH circular), and the axial line (white) to the north pole (RH circular). Thus each circle corresponds to a point on the 2-sphere, defining the Hopf fibration.

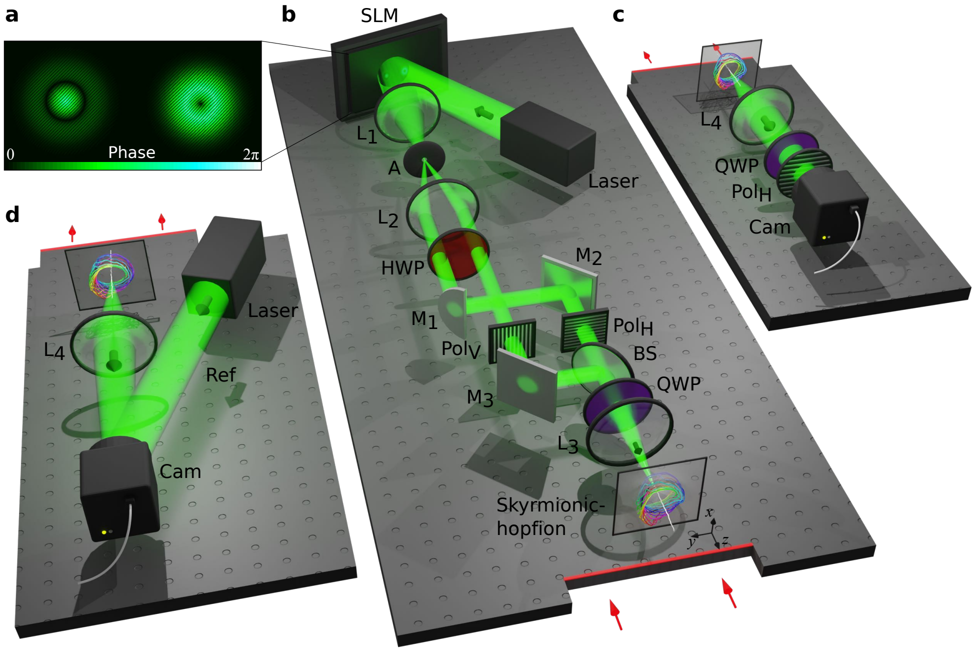

Supplementary Fig. 4. **Experimental System based on a spatial light modulator (SLM). a**, The amplitude and phase information is encoded as a phase-only Fourier hologram on the reflective SLM. **b**, A weighted blazed grating transfers it to the first diffraction order of the initial laser beam (expanded and collimated; horizontal linear polarization; wavelength $\lambda =$ 532nm) when it passes the SLM. The first diffraction order is filtered by an aperture A within a 4f-imaging system (lenses L1 and L2). On-axis superposition of the two beams and their orthogonal circular polarization is ensured by an interferometric system (mirrors M1, M2, M3, beam splitter BS) and polarization optics (H/QWP: half/ quarter-wave plate; PolV,H: vertical/horizontal polarizer). A final lens L3 gives access to the far-field, where the skyrmionic hopfion texture is realised. The VFFR of this structure is based on, **c**, Stokes polarimetry and, **d**, interferometry-based phase measurement (Ref: reference beam) with the corresponding data being collected, transversely resolved, by imaging (lens L4) on a camera (Cam). Longitudinal resolution is achieved by digital propagation enabled by the SLM in combination with the Fourier lens L3. Details on realisation are given in Supplementary Informationand and VFFR is in Methods.

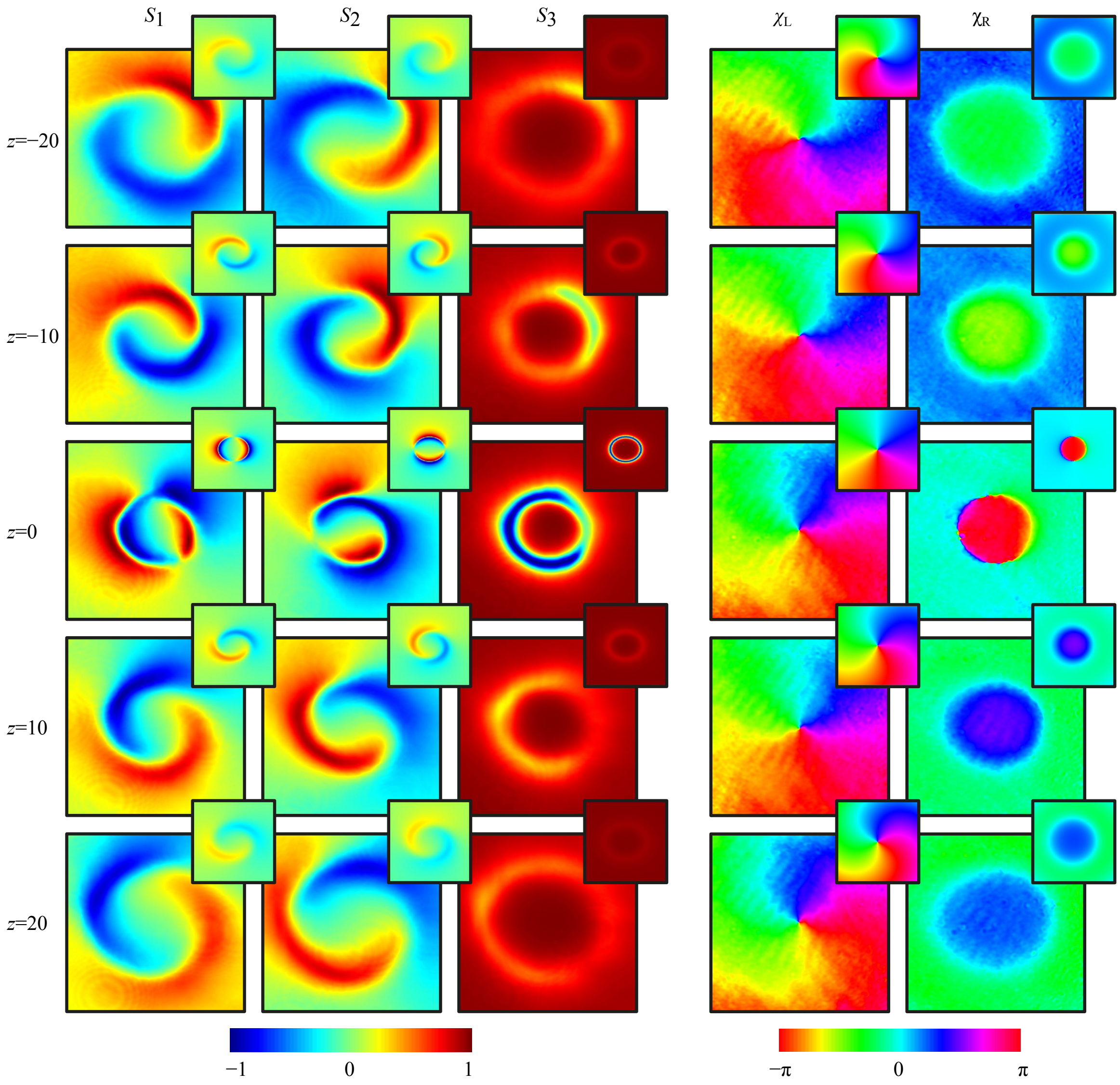

Supplementary Fig. 5. **Experimental measurements.** Transverse planes along the propagation direction ($+z$) of the normalized Stokes parameters S_1, S_2, S_3 and the phase χ_L and χ_R. Within the requirements dictated by topology, the measured beam is consistent with the theoretical predictions shown in the insets. The measured planes show z in mm units.

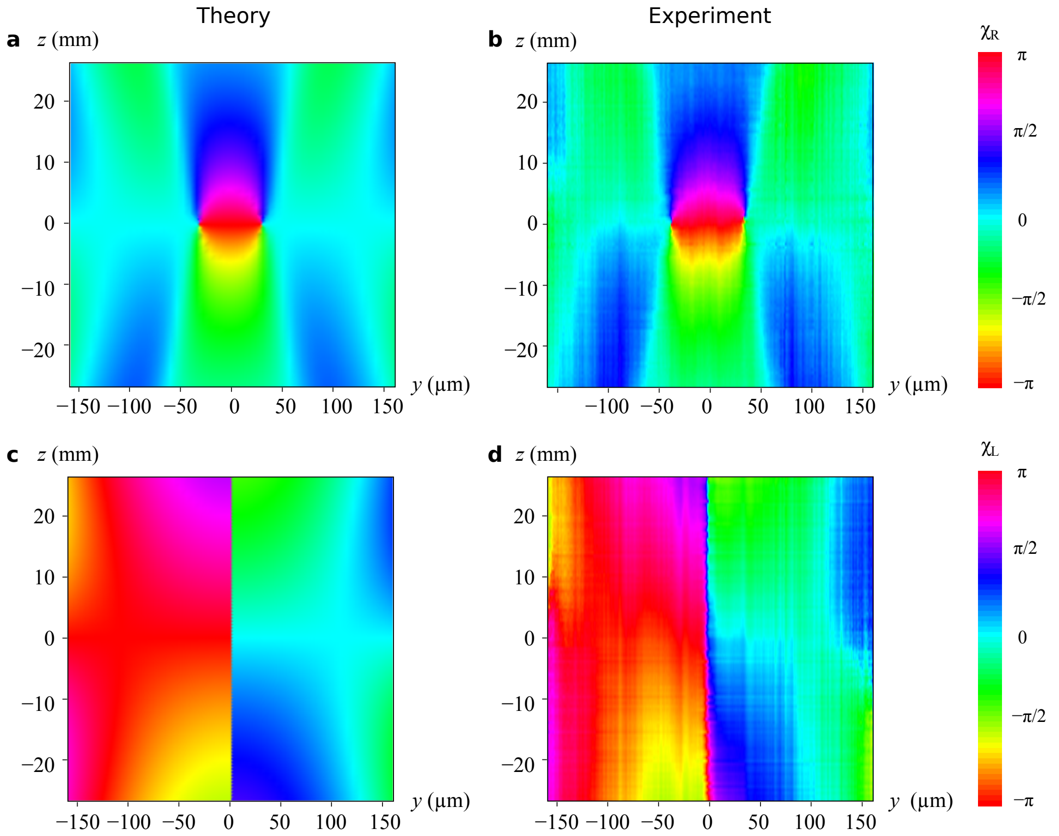

Supplementary Fig. 6. **Comparison of simulated and experimental phase distributions.** **a**, **b**, $\chi_{\mathrm{R}} = \arg E_{\mathrm{R}}$; **c**, **d**, $\chi_{\mathrm{L}} = \arg E_{\mathrm{L}}$. The displayed plane is longitudinal, with $x = 0$.

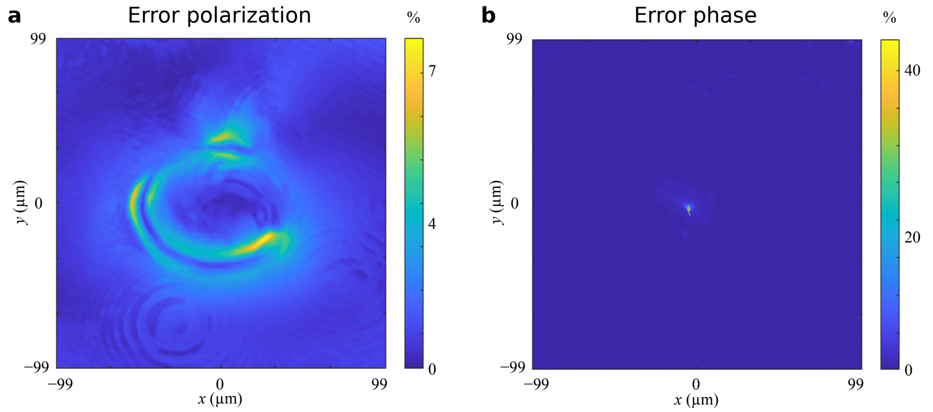

Supplementary Fig. 7. **Density plot of standard deviation a**, for polarization component S_3 and **b**, for LH phase χ_L at the transverse plane $z = 0$. The basis for this errors are ten measurements of the named parameter in the focal plane. A spatial variation is visible, where the error for S_3 is more confined around the nodal line of E_{R} and the error for χ_L around the singularity on the optical axis.

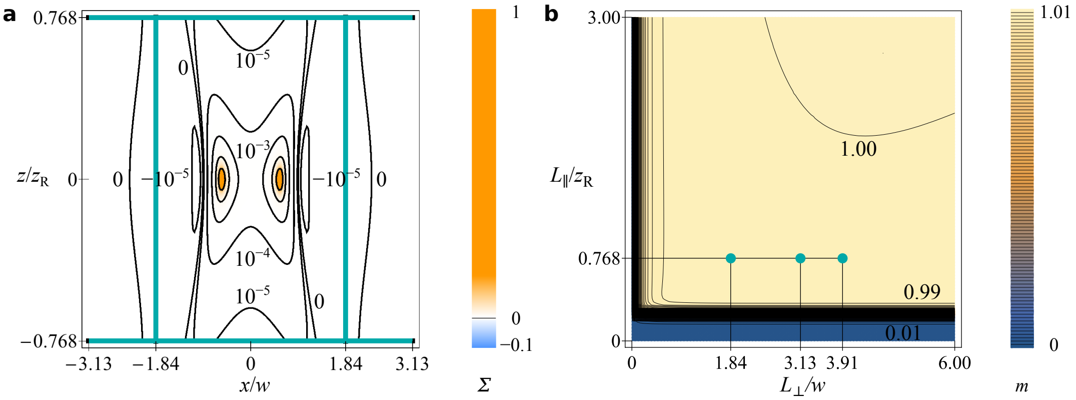

Supplementary Fig. 8. **Skyrme density and its integral.** **a**, Skyrme density of the model of the experimental field across the x, z plane. Most of the density is concentrated around the LH C line ring (as shown equivalently in Supplementary Fig. 10b inset), but contours of very low density reveal other structure. The cyan lines indicate the limits of the evaluation of the Skyrme density of the experimental field. Scale bar is with respect to the maximum density of $\sim 6 \times 10^{11}\,\mathrm{m}^{-3}$. **b**, Integral of Skyrme density in a cube of size $|x| < L_\perp$, $|y| < L_\perp$ and $|z| \leq L_\parallel$. Provided the integration cube includes most of the orange structure as shown in (a), i.e. $L_\parallel \gtrsim 0.1 z_R$, $L_\perp \gtrsim R_0$, then most of the integration volume sizes give 0.99, indicative of the particle-like nature of the configuration.

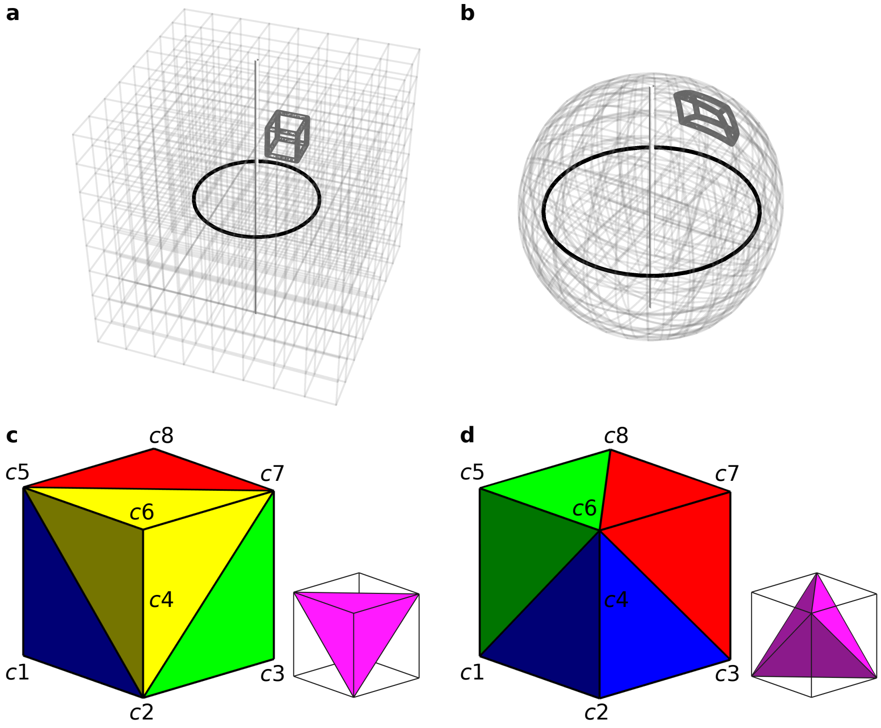

Supplementary Fig. 9. **Mapping of discrete cubic lattice into the 3-sphere.** **a**, Real space C line skeleton of ideal skyrmion field (u, v) of equation (S2.7) overlayed by a cubic lattice in x, y, z, whose vertices represent measurement points, from -2 to 2 at each 0.5. The experimentally measured lattice has much greater resolution. **b**, Image of cubic lattice in the optical hypersphere with $u = E_R$, $v = E_L$. The hypersphere is represented in volume-preserving projection with center corresponding to $u = -1, v = 0$. This is the same projection used in main text Fig. 4b. **c**, **b**, A cubic cell is divided into five tetrahedra with the same vertices: four tetrahedra at corners of the cube, and an internal tetrahedron whose edges are diagonals of the cube faces. The two (mirror image) ways this can be done are shown. For the cubic lattice, alternating between **a** and **b** guarantees neighbouring tetrahedra always share faces (i.e. giving a 3D cell complex).

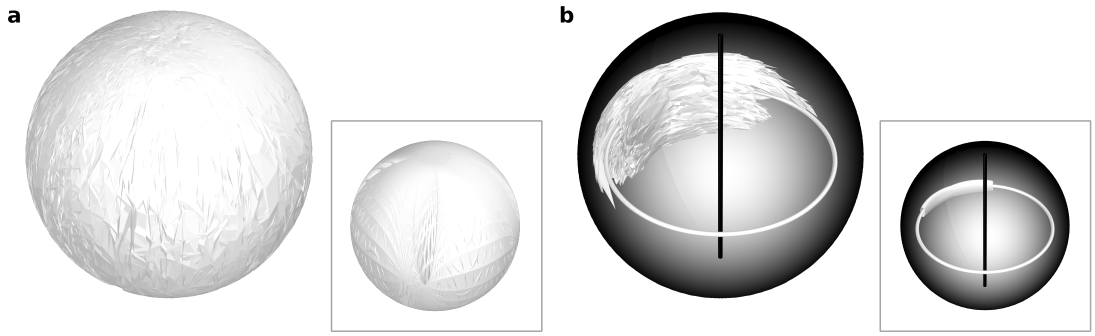

Supplementary Fig. 10. **The experimental Skyrme number represented as a volume in the optical hypersphere.** The optical hypersphere in these plots is represented by a ball with volume-preserving projection, projecting from different points in **a** and **b**. In each case, the boundary of the measured volume in real space (cube) is mapped to the optical hypersphere as the white surface. The inset shows the corresponding surface for the theoretical model field. The volume bounded by this surface, 94.5% of the total volume, corresponding to the hypersphere volume that is covered experimentally, represents the Skyrme number of 0.945. Insets show the theoretical counterpart, covering 99.7% of the 3-sphere volume. **a**, The central point being the polarization state at the focal point. Most of the ball is filled, with the white surface extending almost to the outer surface. **b**, The projection is now from a LH polarization state. The real space axial C line maps to the white loop, and the white surface encloses the complement of the filled volume.

Supplementary Note 1: Parametrising the Optical Hypersphere

In this note we discuss the parameters describing the optical state. Starting with the Stokes parameters and Poincaré sphere for polarization, we generalise by including phase to the 3D parameter space we call the optical hypersphere. This is the unit 3-sphere in 4-dimensional space. Its natural parametrisation by the polarization and phase parameters defines the Hopf fibration structure. The Poincaré sphere is recovered from the optical hypersphere as the 2-sphere base space of the Hopf fibration.

The state of light at each point in a transverse, monochromatic light beam, travelling in the z-direction, is specified by

$$\mathbf{E} = E_\mathrm{R}\widehat{\mathbf{e}}_\mathrm{R} + E_\mathrm{L}\widehat{\mathbf{e}}_\mathrm{L}, \tag{S1.1}$$

in terms of the right- and left-handed (RH, LH) circular polarization complex unit vectors

$$\widehat{\mathbf{e}}_\mathrm{R} = \tfrac{1}{\sqrt{2}}(\widehat{\mathbf{x}} + \mathrm{i}\,\widehat{\mathbf{y}}), \qquad \widehat{\mathbf{e}}_\mathrm{L} = \tfrac{1}{\sqrt{2}}(\widehat{\mathbf{x}} - \mathrm{i}\,\widehat{\mathbf{y}}). \tag{S1.2}$$

Throughout this work, for mathematical convenience, we assume that the field $\mathbf{E}$ is normalized, $|E_\mathrm{R}|^2 + |E_\mathrm{L}|^2 = 1$. Equation (1) of the main text describes E_R and E_L in terms of three angle parameters α, β and γ,

$$E_\mathrm{R} = \cos\tfrac{\beta}{2}\,\mathrm{e}^{\mathrm{i}(\gamma-\alpha)/2}, \qquad E_\mathrm{L} = \sin\tfrac{\beta}{2}\,\mathrm{e}^{\mathrm{i}(\gamma+\alpha)/2}.$$

We begin by describing the standard Stokes parameters and Poincaré sphere formalism, and also describe the phase parameter. This leads to a discussion of 3-sphere geometry and the Hopf fibration, which is then related to the parametrisation of equation (S1.1) using the angles α, β, γ.

1.1. Optical state parameters

1.1.1. Stokes parameters

The (normalized) Stokes parameters S_1, S_2, S_3 may be defined from $\mathbf{E}$ in terms of Pauli spin matrices in the circular basis,

$$\left.\begin{aligned}
S_1 &= \mathbf{E}^*\boldsymbol{\sigma}_1\mathbf{E} = 2\,\mathrm{Re}(E_\mathrm{R}^* E_\mathrm{L}) = 2\left[(\mathrm{Re}\,E_\mathrm{R})(\mathrm{Re}\,E_\mathrm{L}) + (\mathrm{Im}\,E_\mathrm{R})(\mathrm{Im}\,E_\mathrm{L})\right],\\
S_2 &= \mathbf{E}^*\boldsymbol{\sigma}_2\mathbf{E} = 2\,\mathrm{Im}(E_\mathrm{R}^* E_\mathrm{L}) = 2\left[(\mathrm{Re}\,E_\mathrm{R})(\mathrm{Im}\,E_\mathrm{L}) - (\mathrm{Im}\,E_\mathrm{R})(\mathrm{Re}\,E_\mathrm{L})\right],\\
S_3 &= \mathbf{E}^*\boldsymbol{\sigma}_3\mathbf{E} = |E_\mathrm{R}|^2 - |E_\mathrm{L}|^2 = (\mathrm{Re}\,E_\mathrm{R})^2 + (\mathrm{Im}\,E_\mathrm{R})^2 - (\mathrm{Re}\,E_\mathrm{L})^2 - (\mathrm{Im}\,E_\mathrm{L})^2,
\end{aligned}\right\} \tag{S1.3}$$

where $\boldsymbol{\sigma}_1 = \left(\begin{smallmatrix}0&1\\1&0\end{smallmatrix}\right), \boldsymbol{\sigma}_2 = \left(\begin{smallmatrix}0&-\mathrm{i}\\\mathrm{i}&0\end{smallmatrix}\right)$, and $\boldsymbol{\sigma}_3 = \left(\begin{smallmatrix}1&0\\0&-1\end{smallmatrix}\right)$. These quadratic combinations of the four fundamental real-valued field quantities, $\mathrm{Re}\,E_\mathrm{R}, \mathrm{Im}\,E_\mathrm{R}, \mathrm{Re}\,E_\mathrm{L}, \mathrm{Im}\,E_\mathrm{L}$, only involve information about the polarization state – they are independent of the overall phase, typical of measurable quantum expectation values and classical constants of the motion. With these definitions, it is not difficult to show that $S_1^2 + S_2^2 + S_3^2 = 1$. The resulting normalized 3-vector (S_1, S_2, S_3), the Stokes vector, lies on the surface of a unit 2-sphere, the Poincaré sphere [1, 2]. Every polarization state (regardless of its phase) corresponds to a point on this sphere: RH circular ($E_\mathrm{L} = 0$) to the north pole $(0, 0, +1)$, LH circular ($E_\mathrm{R} = 0$) to the south pole $(0, 0, -1)$, linearly polarized states ($S_3 = 0$) to the equator, and so on. More general elliptical polarized states with RH (LH) circulation, with $S_3 > 0$ ($S_3 < 0$) occur in the northern (southern) hemisphere. This parametrisation of elliptic states on the Poincaré sphere is demonstrated in main text Fig. 1.

The three standard Stokes parameters description may be extended by two extra, alternative parameters (mathematically defined in a similar way to S_1 and S_2) [3, 4] which determine the phase information,

$$\left.\begin{aligned}
S_4 &= \mathrm{Re}(E_\mathrm{R} E_\mathrm{L}) = 2\left[(\mathrm{Re}\,E_\mathrm{R})(\mathrm{Re}\,E_\mathrm{L}) - (\mathrm{Im}\,E_\mathrm{R})(\mathrm{Im}\,E_\mathrm{L})\right],\\
S_5 &= \mathrm{Im}(E_\mathrm{R} E_\mathrm{L}) = 2\left[(\mathrm{Re}\,E_\mathrm{R})(\mathrm{Im}\,E_\mathrm{L}) + (\mathrm{Im}\,E_\mathrm{R})(\mathrm{Re}\,E_\mathrm{L})\right].
\end{aligned}\right\} \tag{S1.4}$$

With S_3, these also satisfy the normalization condition $S_3^2 + S_4^2 + S_5^2 = 1$. This means that an alternative sphere similar to the Poincaré sphere can be defined with cartesian coordinates S_3, S_4, S_5 instead of S_1, S_2, S_3 [3, 4]. We will discuss the significance of this definition below.

1.1.2. Polarization ellipse and phase

Apart from the overall normalization, the optical state $\mathbf{E}$ is determined by the normalized, complex two-component electric vector $(E_\mathrm{R}, E_\mathrm{L})$, or equivalently, the normalized 4-dimensional vector $(\mathrm{Re}\,E_\mathrm{R}, \mathrm{Im}\,E_\mathrm{R}, \mathrm{Re}\,E_\mathrm{L}, \mathrm{Im}\,E_\mathrm{L})$. These parameters, together with the equivalent angles α, β, γ used in the main text and defined in equation (1), are related to the geometry of the polarization ellipse. The time-dependent, monochromatic electric field $\mathbf{E}^\mathrm{Re}$ traces out an ellipse in the xy-plane in time t with period $2\pi/\omega$:

$$\begin{aligned} \mathbf{E}^\mathrm{Re}(\omega t) &= \mathrm{Re}\{\mathbf{E}\,\mathrm{e}^{-\mathrm{i}\omega t}\} \qquad \text{(S1.5)} \\ &= \tfrac{1}{\sqrt{2}}\{[\mathrm{Re}(E_\mathrm{R}+E_\mathrm{L})\cos\omega t + \mathrm{Im}(E_\mathrm{R}+E_\mathrm{L})\sin\omega t]\,\hat{\mathbf{x}} + [\mathrm{Im}(E_\mathrm{L}-E_\mathrm{R})\cos\omega t + \mathrm{Re}(E_\mathrm{R}-E_\mathrm{L})\sin\omega t]\,\hat{\mathbf{y}}\}\,. \end{aligned}$$

A simple way of visualising this ellipse is in terms of its major axis $\mathbf{a}$ and minor axis $\mathbf{b}$; the normalization requires $|\mathbf{a}|^2 + |\mathbf{b}|^2 = 1$ and the axes are perpendicular, $\mathbf{a}\cdot\mathbf{b} = 0$. The ellipse curve traced out by $\mathrm{Re}\{(\mathbf{a}+\mathrm{i}\,\mathbf{b})\,\mathrm{e}^{-\mathrm{i}\omega t}\}$ is the same as that traced out by $\mathbf{E}^\mathrm{Re}(\omega t)$; the only difference is the starting point on the ellipse, which is related to the phase parameter γ.

The phase of $\mathbf{E}$ is related to the overall phase factor at $t = 0$ multiplying the complex combination of major and minor axis vectors at time $t = 0$, i.e. $\mathbf{E} = \mathrm{e}^{\mathrm{i}\gamma/2}(\mathbf{a}+\mathrm{i}\,\mathbf{b})$. From the discussion above, this implies that $\mathbf{E}^\mathrm{Re}(\omega t = \gamma/2) = \mathbf{a}$. Thus the effective phase evolves with time. It is necessary to be careful with this definition, as described in the following.

The major and minor axes correspond, with respect to t, to maxima and minima of

$$|\mathbf{E}^\mathrm{Re}(\omega t)|^2 = \tfrac{1}{2}\left[1 + S_4\cos(2\omega t) + S_5\sin(2\omega t)\right], \qquad \text{(S1.6)}$$

which has been simplified using the alternative Stokes parameters (S1.4).

This is a maximum at $\omega t_\mathrm{max} = \gamma_+$ and a minimum at $\omega t_\mathrm{min} = \gamma_-$, where

$$\gamma_+ \equiv \tfrac{1}{2}\arctan(S_4, S_5) = \tfrac{1}{2}\arg(E_\mathrm{R}E_\mathrm{L}), \qquad \gamma_- \equiv \tfrac{1}{2}\arctan(-S_4, -S_5). \qquad \text{(S1.7)}$$

Since arctan is a periodic function with period π, there is another maximum/minimum at $\gamma_\pm + \pi$. We therefore have

$$\mathbf{a} = \mathbf{E}^\mathrm{Re}(\gamma_+), \qquad \mathbf{b} = \mathbf{E}^\mathrm{Re}(\gamma_-). \qquad \text{(S1.8)}$$

In fact, these give a simple expression for the original complex vector $\mathbf{E}$,

$$\mathbf{E} = (\mathbf{a}+\mathrm{i}\,\mathbf{b})\,\mathrm{e}^{\mathrm{i}\gamma_+}, \qquad \text{(S1.9)}$$

where γ_+ determines the phase factor multiplying the complex vector of major and minor axes, with a π ambiguity. The definition of the phase parameter γ correctly over the 4π range, i.e. as $2\gamma_+$ or $2\gamma_+ \pm 2\pi$, requires some care, as described below. With this in mind, we discuss each of the angles α, β, γ used in the main text and defined in main text equation (2).

1.1.3. Polarization azimuth α and ellipticity β

The angle α is defined as the azimuth angle on the Poincaré sphere,

$$\alpha = \arctan(S_1, S_2) = \arg(E_\mathrm{R}^* E_\mathrm{L}). \qquad \text{(S1.10)}$$

As shown in Supplementary Fig. 1, $\alpha/2$ is the angle between the polarization ellipse major axis and the x-direction, i.e. $\mathbf{a} = |\mathbf{a}|\left(\cos(\tfrac{1}{2}\alpha)\hat{\mathbf{x}} + \sin(\tfrac{1}{2}\alpha)\hat{\mathbf{y}}\right)$. This follows from equation (S1.8), after simplification and substituting the expression for the maximum γ_+ from equation (S1.7) into (S1.5).

This half angle cannot distinguish between $\pm\mathbf{a}$: the orientation angle $\alpha/2$ of the major axis of the ellipse is in fact a director, only defined over a π range, say $-\pi/2 < \alpha/2 \leq \pi/2$, consistent with our definition of the range $-\pi < \alpha \leq \pi$. This half-angle nature of α implies that the azimuth angle on the Poincaré sphere changes twice as quickly as the ellipse orientation angle in real space, and has been subject to much comment in the literature. The factor of 1/2 on α in equation (2) is naturally associated with spinorial parametrisation. Its range from $-\pi$ to π is consistent with its definition as the argument of the product $E_\mathrm{R}^* E_\mathrm{L}$.

The angle β is the polar angle on the Poincaré sphere,

$$\beta = \arccos S_3, \qquad \sin\beta = \sqrt{S_1^2 + S_2^2} = \sqrt{S_4^2 + S_5^2}. \qquad \text{(S1.11)}$$

It determines the shape and the handedness of the ellipse, and it can also be shown that

$$\cos\beta \;=\; 2\hat{\mathbf{z}}\cdot(\mathbf{a}\times\mathbf{b}) \;=\; 2|\mathbf{a}||\mathbf{b}|\,\mathrm{sign}\,S_3. \tag{S1.12}$$

Therefore, $\cos\beta$ is proportional to the area of the bounding rectangle of the ellipse, with the normalization condition $|\mathbf{a}|^2+|\mathbf{b}|^2=1$ corresponding to fixing the main diagonal of the bounding rectangle, as shown in Supplementary Fig. 1. Since $-1\leq S_3\leq 1$, the range of β is from 0 to π.

1.1.4. Optical phase and γ

The definition above of the phases $\gamma_\pm$ at the maxima and minima ignored the subtlety that we did not distinguish between $+\mathbf{a}$ and $-\mathbf{a}$. In our previous discussion, we saw that this only determines the ellipse major axis as a director $\pm\mathbf{a}$, and the azimuth parameter α (with $-\pi<\alpha\leq\pi$) is twice the angle between the major axis and $\hat{\mathbf{x}}$.

In the definition of γ_+ in equation (S1.7), the choice of $\gamma_+=\frac{1}{2}\arg(E_\mathrm{R}E_\mathrm{L})$ rather than $\frac{1}{2}\arg(E_\mathrm{R}E_\mathrm{L})+\pi$ was arbitrary, and is defined $-\pi/2<\gamma_+\leq\pi/2$. This choice represents the phase difference between the point on $\mathbf{E}^\mathrm{Re}(0)$ on the ellipse and the closer of the two major axis vectors $\pm\mathbf{a}$. As the phase cycles around the whole ellipse, which one of $\pm\mathbf{a}$ is closer to $\mathbf{E}^\mathrm{Re}(\gamma/2)$ jumps, as $\mathbf{E}^\mathrm{Re}$ crosses the minor axis $\pm\mathbf{b}$ when $\gamma_+=\pm\pi/2$.

To determine the phase value unambiguously, it is necessary to fix a choice of major axis vector $\pm\mathbf{a}$ for each polarization state, say $+\mathbf{a}$. This allows the phase position of $\mathbf{E}^\mathrm{Re}(0)$ to be determined with respect to $\mathbf{a}$, which extends the range of γ_+ to a full cycle, $-\pi<\gamma_+\leq\pi$.

Just as the angles α and β are defined as the half-angles of the natural physical angle, so is the phase parameter γ twice the value of the physical phase angle, $\gamma=2\gamma_+$. This implies that γ varies over a 4π range, $-2\pi<\gamma\leq 2\pi$, with the general definition

$$\gamma=\arg E_\mathrm{R}+\arg E_\mathrm{L}. \tag{S1.13}$$

In practice significant care is needed numerically to determine the correct branch cut of γ when $\mathbf{E}^\mathrm{Re}$ crosses $-\mathbf{a}$. A numerical value for γ can be determined nonlocally for instance by tracing the evolution of $\mathbf{E}^\mathrm{Re}$ with respect to the chosen sign of the major axis vector.

The normalized polarization state $(E_\mathrm{R},E_\mathrm{L})$ resembles a 2-component spinor with phase. These are often used to represent spatial orientations and rotations (e.g. using the Cayley-Klein parametrisation of Euler angles [5]), parametrising the special orthogonal group SO(3). On the other hand, the full state of light, being parameterised by the 3-sphere, corresponds to a point in the special unitary group SU(2), which is the double cover of SO(3) (i.e. for every point in SO(3) there are two in SU(2)). This double covering, in the α,β,γ parametrisation, corresponds to the 2π versus 4π range of γ [6].

1.1.5. The Poincaré sphere and its projections

The approach to polarization and the optical state $(E_\mathrm{R},E_\mathrm{L})$ in the circular bases is very reminiscent of the mathematical spinor formalism. This is most familiar in the representation of the state of a quantum spin 1/2 particle, where the spin direction in 3D real space is represented by a point on the Bloch sphere.

Physically, a monochromatic electric field has spin 1. Since we only consider a 2-component transverse field, the complex field $(E_\mathrm{R},E_\mathrm{L})$ mathematically represents a normalized complex spinor. The optical state is not truly a spin 1/2 system, therefore, and so $(E_\mathrm{R},E_\mathrm{L})$ is properly a pseudospinor. The corresponding Poincaré sphere is defined in an abstract 3D space, unlike the Bloch sphere, whose directions correspond to those in 3D real space.

An alternative representation of polarization states is via the complex ratio

$$\Psi=\frac{E_\mathrm{L}}{E_\mathrm{R}}=\tan(\beta/2)\,\mathrm{e}^{\mathrm{i}\,\alpha}=\rho(\beta)\,\mathrm{e}^{\mathrm{i}\,\alpha}, \tag{S1.14}$$

which defines a point in the complex plane with modulus (radius) $\tan(\beta/2)$ and azimuth α. This point corresponds to the stereographic projection of the Stokes vector, centred at the north pole, and including the point in the plane at infinity ($\beta=\pi$, i.e. the south pole), the plane is topologically equivalent to the sphere. This is a direct illustration of one-point compactification: a euclidean plane, with a single point ∞ adjoined, becomes, topologically, identical to a sphere. This procedure can be generalised directly to euclidean n-space and the n-sphere.

In principle, any positive monotonic function $\rho(\beta)$ can be used as a radial coordinate to map the 2-sphere to the plane; when $\rho(\beta=\pi)$ is finite, the map is in a disk of finite radius, and all points on the circumference $\rho(\pi)$ correspond to the south pole. In the example of stereographic coordinates above, $\rho=\tan(\beta/2)$; this projection from the sphere to the

plane is conformal, preserving angles. Another important example is $\rho(\beta) = 2\cos(\beta)$, Lambert equal area projection, where areas on the plane correspond to areas on the sphere. Areas are preserved here even though the south pole $\beta = \pi$ projects to a circle (radius 2).

The stereographic representation (S1.14) is based on a north pole projection, which associates the north pole $\beta = 0$ with the origin of the plane, and the south pole $\beta = \pi$ with ∞. With this choice, positive orientation (the local right-handed sense on the sphere) is consistent with the orientation of the increase of the sphere azimuth coordinate α. We could instead have chosen south pole projection where $\rho = \cot(\beta/2)$, which associates the south pole with the origin and the north pole with ∞. For this projection to preserve orientation, positive orientation is associated with a decrease in sphere azimuth angle α, so the complex coordinate associated with orientation-preserving south pole projection is $\cot(\beta/2)\,\mathrm{e}^{-\mathrm{i}\alpha}$. This is important for the analogous argument for the 3-sphere.

1.2. The hypersphere and the Hopf fibration

1.2.1. Parametrising the hypersphere

Many of the details discussed above about 2-sphere geometry, topology and parametrisations generalise directly to the 3-sphere (hypersphere) [7, 8]. For instance, we can consider 4-dimensional (4D) space with cartesian coordinates W, Z, X, Y. The reason for the non-standard ordering is as follows.

The 3-sphere is defined by all possible 4D unit vectors, represented using overarrow $\vec{n} = (n_W, n_Z, n_X, n_Y)$. Points on the 3-sphere can be parameterised by hyperspherical polar angles ω, θ, φ with $0 \le \omega, \theta \le \pi$, $-\pi < \varphi \le \pi$ in the following way,

$$\left.\begin{aligned} n_W &= \cos\omega, \\ n_Z &= \sin\omega\cos\theta, \\ n_X &= \sin\omega\sin\theta\cos\varphi, \\ n_Y &= \sin\omega\sin\theta\sin\varphi. \end{aligned}\right\} \tag{S1.15}$$

Clearly these satisfy $n_W^2 + n_Z^2 + n_X^2 + n_Y^2 = 1$. (Note that the 4-space azimuthal angle φ is different from the 3D real space azimuthal angle ϕ used later.)

Features of the topology of the 3-sphere can also be discerned from this parametrisation, by relating the XYZ coordinates to the 3D cartesian coordinates. n_X, n_Y, n_Z resemble the coordinates of a 2-sphere, parameterised by spherical polar angles θ and φ as normal, and with radial coordinate $\sin\omega$: all these spheres enclose a central point (at which $\omega = 0$), and extend out to a sphere of unit radius at $\omega = \pi$. In this embedding, the boundary sphere corresponds to the single point $\omega = \pi$ in the 3-sphere (similar to the embedding of the 2-sphere in the 2-plane as discussed above). This solid ball in 3D is therefore a projection of the 3-sphere into euclidean 3D space, for which $\sin\omega = \rho(\omega)$ as defined in Sec. 1.1, related to the quaternion representation of rotations (where the angle of rotation is 2ω) [9, 10].

Just as for the 2-sphere, we can consider different ball projections of the 3-sphere into 3D euclidean space by different choices of $\rho(\omega)$. For instance, stereographic projection to X, Y, Z coordinates is achieved via $\rho(\omega) = \tan\frac{1}{2}\omega$. Explicitly, this gives

$$n_X = \frac{2X}{1+X^2+Y^2+Z^2}, \quad n_Y = \frac{2Y}{1+X^2+Y^2+Z^2}, \quad n_Z = \frac{2Z}{1+X^2+Y^2+Z^2}, \quad n_W = \frac{1-X^2-Y^2-Z^2}{1+X^2+Y^2+Z^2}. \tag{S1.16}$$

As in 2D stereographic projection (S1.14), the 3-sphere point $\omega = \pi$ corresponds in euclidean 3D space to the point ∞. We can also write complex coordinates U, V for the 3-sphere,

$$\left.\begin{aligned} U(X,Y,Z) &= n_W + \mathrm{i}\,n_Z = (1-X^2-Y^2-Z^2+2\,\mathrm{i}\,Z)/(1+X^2+Y^2+Z^2), \\ V(X,Y,Z) &= n_X + \mathrm{i}\,n_Y = 2(X+\mathrm{i}\,Y)/(1+X^2+Y^2+Z^2), \end{aligned}\right\} \tag{S1.17}$$

where in each case the second equality corresponds to stereographic projection to euclidean space (with the point with $\omega = \pi$, $n_W = -1$ at infinity), and $|U|^2 + |V|^2 = 1$. The modulus and phase of U are shown in Supplementary Fig. 2a; as described below, with φ these correspond to toroidal coordinates in euclidean 3-space.

Stereographic projection provides a very natural visualisation of the hypersphere in terms of familiar, infinite 3D euclidean space. Yet other projections of the 3-sphere into euclidean 3-space are possible [9, 10]. The representations of the optical hypersphere in main text Fig. 4, are chosen to preserve 3D volume, analogous to Lambert equal area projection, for which the radius function is $\rho(\omega) = [\frac{3}{4}(2\omega - \sin 2\omega)]^{1/3}$ [9].

1.2.2. Hopf fibration

We have considered the polar angle parametrisation of the hypersphere using ω, θ, φ by analogy with the polar angles of the 2-sphere. They are not the only natural parameters of the 3-sphere, and in fact are not the natural physical parametrisation of the optical hypersphere.

We identify the complex scalar U with the RH component of the optical field, i.e. $U = E_\mathrm{R}$, and V with the LH component, $V = E_\mathrm{L}$; this is possible since both pairs of complex scalars are normalized, $1 = |U|^2 + |V|^2 = |E_\mathrm{R}|^2 + |E_\mathrm{L}|^2$. This identification gives the optical state parameters α, β, γ as the parameters of the Hopf fibration in the optical hypersphere, described in the following.

Firstly, the identification of U and V with E_R and E_L allows us to find the relationship between α, β, γ and θ, φ, ω,

$$\left.\begin{array}{rcccl} E_\mathrm{R} &=& \cos(\beta/2)\,\mathrm{e}^{\mathrm{i}(\gamma-\alpha)/2} &=& \cos\omega + \mathrm{i}\sin\omega\cos\theta, \\ E_\mathrm{L} &=& \sin(\beta/2)\,\mathrm{e}^{\mathrm{i}(\gamma+\alpha)/2} &=& \sin\omega\sin\theta\,\mathrm{e}^{\mathrm{i}\varphi}. \end{array}\right\} \tag{S1.18}$$

The unit 4-vector $\vec{n}$ is identified with the 4-vector of real and imaginary parts of E_R and E_L considered earlier,

$$\begin{aligned} \vec{n} &= (\mathrm{Re}\,E_\mathrm{R}, \mathrm{Im}\,E_\mathrm{R}, \mathrm{Re}\,E_\mathrm{L}, \mathrm{Im}\,E_\mathrm{L}) \\ &= \left(\cos\tfrac{1}{2}\beta\cos\tfrac{1}{2}[\gamma-\alpha], \cos\tfrac{1}{2}\beta\sin\tfrac{1}{2}[\gamma-\alpha], \sin\tfrac{1}{2}\beta\cos\tfrac{1}{2}[\gamma+\alpha], \sin\tfrac{1}{2}\beta\sin\tfrac{1}{2}[\gamma+\alpha]\right) \\ &= (\cos\omega, \sin\omega\cos\theta, \sin\omega\sin\theta\cos\varphi, \sin\omega\sin\theta\sin\varphi)\,. \end{aligned} \tag{S1.19}$$

We now interpret α, β and γ in the 3-sphere, represented by euclidean 3D space under stereographic projection. This is possible since the Hopf fibration of the 3-sphere can in fact be constructed directly from toroidal coordinates [11–13] in euclidean 3-space, considered as the stereographic projection of the 3-sphere.

Consider the complex coordinates U, V as complex scalar functions of 3D euclidean space. U has a circular nodal line (phase singularity) of unit radius in the XY-plane centred on the origin, which we call the reference circle (anticipating the use of toroidal coordinates below). This is threaded by a nodal line in V along the Z-axis (which corresponds to a circle in the 3-sphere, with one point mapping to ∞ in euclidean 3-space), which we call the axial line. As a stereographic representation of the optical hypersphere, the states of LH polarization ($\beta = 0$) occur on the reference circle, and the states of RH polarization ($\beta = \pi$) on the axial line (this includes the point at ∞, closing topologically into a loop). The phase varies smoothly along these lines. Representing the RH and LH states of polarization, the axial line and reference circle are special, as curves in the 3D optical hypersphere, corresponding to the points of circular polarization at the poles of the 2D Poincaré sphere.

We now construct the Hopf structure using the fact that U and V are naturally related to toroidal coordinates φ, σ, τ in X, Y, Z space [11–13]. The toroidal coordinate φ is the familiar azimuth angle of spherical and cylindrical polar coordinates,

$$\varphi \;=\; \arctan(X, Y) \;=\; \arg V \;=\; \tfrac{1}{2}(\gamma+\alpha), \tag{S1.20}$$

from equations (S1.17), and (S1.18). We use the standard convention of increase of φ, but note it needs to be reversed when considering 3D orientation. The coordinate σ is the poloidal angle about the reference circle $U = 0$ (also see Supplementary Fig. 2a), and in fact, also from equations (S1.18) and (S1.17),

$$\sigma \;=\; \arctan(X^2+Y^2+Z^2-1, 2\,\mathrm{i}\,Z) \;=\; \arg U \;=\; \tfrac{1}{2}(\gamma-\alpha). \tag{S1.21}$$

On the punctured plane $Z = 0, R > 0$, $\sigma = 0$, and σ increases around the reference circle in a RH sense in a plane of fixed φ, as in Supplementary Fig. 2a. The final toroidal coordinate, τ, is defined over the range $0 \leq \tau < \infty$, by

$$\cosh\tau \;=\; \frac{X^2+Y^2+Z^2+1}{\sqrt{(X^2+Y^2+Z^2)^2+1-2(X^2+Y^2-Z^2)}} \;=\; |U|^{-1} \;=\; \sec\tfrac{1}{2}\beta \;=\; \sqrt{\frac{2}{1+\cos\beta}}, \tag{S1.22}$$

which is simply a monotonic function of β. Surfaces of constant τ are tori centred on the axial line which enclose the reference circle, with major radius $\coth\tau$ and minor radius $\mathrm{cosech}\,\tau$. $\tau = 0$ on the axial line, with $\tau \to \infty$ on the reference circle.

Now, the azimuthal and poloidal angles φ and σ are angles winding around the tori of constant τ in flat planes; their sum and difference, $\alpha = \varphi - \sigma$ and $\gamma = \varphi + \sigma$, both wind around the tori as $(1, 1)$ torus curves. Loci of constant β and α, therefore give curves—in fact Villarceau circles—which wrap around both nodal lines in U, V as γ. The resulting structure of lines on which α, β are constant define the Hopf fibration, as in Supplementary Fig. 3. Position on these Hopf circles is parameterised by γ on its 4π cycle.

The Hopf fibration represents the 3-sphere as a fiber bundle, whose fibers are the Villarceau circles for fixed α, β along which γ varies. The parameters β, α themselves are determined by points on the Poincaré 2-sphere (the base space of the fibration). The loci of constant β (i.e. constant S_3) correspond to constant τ, i.e. tori, around which are the nodal line loops corresponding to $\beta = 0, \pi$.

The choice of α, β parametrising the base space and γ parametrising the fibers is only one possibility; it is also possible to parametrise the fibers with α, in which case the base space is the sphere parameterised by α, γ (with appropriate modification to the ranges). The sphere of this fibration is via the Hopf map of the alternative Stokes parameters S_3, S_4, S_5 introduced in equation (S1.4).

1.2.3. 3-sphere volume

The unit 3-sphere has total volume (hypersurface area) $2\pi^2$, as we now discuss. The normalized volume element $\mathrm{d}^3\Omega$ of the hypersphere can be derived from the hypersurface area of the 3-sphere in 4D space, in terms of either set of angle coordinates θ, φ, ω, or α, β, γ. Some care is needed to ensure the proper sense of orientation on this 3-manifold; we define this in terms of the usual orientation of euclidean 3D space for stereographic coordinates, which corresponds to ordering the angles ω, θ, φ.

In terms of ω, θ, φ, the normalized volume element (dividing by the hypersphere volume $2\pi^2$) is

$$\mathrm{d}^3\Omega \;=\; \frac{1}{2\pi^2}\det[\,\vec{n}, \partial_\omega \vec{n}, \partial_\theta \vec{n}, \partial_\varphi \vec{n}]\,\mathrm{d}\omega\,\mathrm{d}\theta\,\mathrm{d}\varphi \;=\; \frac{1}{2\pi^2}\sin^2\omega\sin\theta\,\mathrm{d}\omega\,\mathrm{d}\theta\,\mathrm{d}\varphi, \tag{S1.23}$$

where $\det[\,\vec{n}, \partial_\omega \vec{n}, \partial_\theta \vec{n}, \partial_\varphi \vec{n}]$ denotes the matrix of column vector partial derivatives of $\vec{n}$, calculated using equation (S1.19). This determinant plays the role, in 4D space, of the scalar triple product in 3D. With this normalization, the integral of these angles over the 3-sphere with ranges $0 \le \omega, \theta \le \pi$, $-\pi < \varphi \le \pi$ indeed gives unity.

We can apply this procedure to the optical state parameters α, β, γ. In order to preserve the sign of the determinant (i.e. the 3-sphere orientation), it is necessary to use the ordering γ, β, α (or a cyclic permutation of them), i.e.

$$\mathrm{d}^3\Omega \;=\; \frac{1}{2\pi^2}\det[\,\vec{n}, \partial_\gamma \vec{n}, \partial_\beta \vec{n}, \partial_\alpha \vec{n}]\,\mathrm{d}\gamma\,\mathrm{d}\beta\,\mathrm{d}\alpha \;=\; \frac{1}{16\pi^2}\sin\beta\,\mathrm{d}\gamma\,\mathrm{d}\beta\,\mathrm{d}\alpha \;=\; \frac{1}{16\pi^2}\,\mathrm{d}\alpha\,\mathrm{d}(\cos\beta)\,\mathrm{d}\gamma. \tag{S1.24}$$

In the last equality, β was replaced by $\cos\beta$; the volume element in terms of $\alpha, \cos\beta, \gamma$ has no prefactor, and is positive in lexicographical ordering (since $\cos\beta$ increases as β decreases).

Supplementary Note 2: Designing the optical skyrmionic hopfion

In this note we summarise the theoretical model of the skyrmionic hopfion field. This field configuration captures the Hopf fibration structure in real space, realising all phases and polarizations within the propagation volume. We construct solutions to the (paraxial) optical wave equation that show the desired topology, then we describe how we select the optimal superposition of Laguerre-Gauss modes representing a physically realisable optical skyrmionic hopfion.

Such a field is designed by creating RH and LH fields, as functions of real 3D space using cartesian coordinates x, y, z or cylindrical coordinates R, ϕ, z, i.e. $E_\mathrm{R}(x, y, z)$ and $E_\mathrm{L}(x, y, z)$. We introduce unnormalized forms of the field components $\psi_\mathrm{R}(x, y, z), \psi_\mathrm{L}(x, y, z)$, such that

$$E_\mathrm{R} \;=\; \frac{\psi_\mathrm{R}}{\sqrt{|\psi_\mathrm{R}|^2 + |\psi_\mathrm{L}|^2}}, \qquad E_\mathrm{L} \;=\; \frac{\psi_\mathrm{L}}{\sqrt{|\psi_\mathrm{R}|^2 + |\psi_\mathrm{L}|^2}}. \tag{S2.1}$$

The scalar fields $\psi = \psi_\mathrm{R}, \psi_\mathrm{L}$ are solutions of the paraxial wave equation $\nabla_\perp^2\psi + 2\,\mathrm{i}\,k\partial_z\psi = 0$, where $\nabla_\perp^2 = \partial_x^2 + \partial_y^2$ is the 2D transverse laplacian, $k = 2\pi/\lambda$ is the wavenumber; these are good representations of scalar components of collimated laser beams [14]. For future consideration, we define the phases of the scalar components, $\chi_\mathrm{R} \equiv \arg\psi_\mathrm{R}$, and $\chi_\mathrm{L} \equiv \arg\psi_\mathrm{L}$.

As described in the main text and methods, the skyrmionic hopfion field is designed by a superposition of Laguerre-Gaussian (LG) beams, with the RH component ψ_R being zero on a circle in the focal plane, and ψ_R zero on the beam axis. The zeros of the circular components correspond to C lines, along which the state of polarization is circular [15]. In the full polarization field, these correspond to a RH C line along the axis, and a LH C line circle. The design of the optical skyrmionic hopfion field is thus based on the principle that C lines are the skeleton of the complex optical polarization field, although careful tuning of the superposition is necessary to guarantee the covering of the optical hypersphere within the focal volume.

2.1. Laguerre-Gauss modes

Laguerre-Gauss (LG) modes are a convenient set of basis functions for solutions of the paraxial wave equation. The LG modes are naturally expressed in cylindrical coordinates R, ϕ, z by [14, 16]

$$\mathrm{LG}^{3\mathrm{D}}_{\ell,p}(R,\phi,z;w) = \sqrt{\frac{p!}{\pi(|\ell|+p)!}}\frac{(R/w)^{|\ell|}\mathrm{e}^{\mathrm{i}\ell\phi}}{w}\frac{(1-\mathrm{i}z/z_\mathrm{R})^p}{(1+\mathrm{i}z/z_\mathrm{R})^{p+|\ell|+1}}\mathrm{L}^{|\ell|}_p\left(\frac{(R/w)^2}{1+(z/z_\mathrm{R})^2}\right)\exp\left(-\frac{(R/w)^2}{2(1+\mathrm{i}z/z_\mathrm{R})}\right) \tag{S2.2}$$

$$= \sqrt{\frac{p!}{\pi(|\ell|+p)!}}\frac{R^{|\ell|}\mathrm{e}^{\mathrm{i}\ell\phi}}{w^{|\ell|+1}}\frac{\mathrm{e}^{-R^2/w^2(1+z^2/z_\mathrm{R}^2)}}{(1+(\frac{z}{z_\mathrm{R}})^2)^{|\ell|+1}}\mathrm{L}^{|\ell|}_p\left(\frac{(R/w)^2}{1+(\frac{z}{z_\mathrm{R}})^2}\right)\mathrm{e}^{\mathrm{i}(|\ell|+2p+1)\arctan(z/z_\mathrm{R})}\,\mathrm{e}^{\mathrm{i}\,R^2 z z_\mathrm{R}/w^2(z_\mathrm{R}^2+z^2)}\,. \tag{S2.3}$$

Here, w is the width of the gaussian envelope setting the transverse size of the beam, and $z_\mathrm{R} = kw^2$, the Rayleigh range of the gaussian beam, determines the longitudinal lengthscale. $\mathrm{L}^{|\ell|}_p$ denotes an associated Laguerre polynomial [12, 14]. The positive or negative (or zero) integer ℓ is the azimuthal quantum number; when $\ell \neq 0$, there is an optical vortex along the axis of topological strength ℓ. The nonnegative integer p is the radial order, corresponding to the order of the Laguerre polynomial, giving the number of nodal rings concentric to the axis in a typical transverse plane. The term $\mathrm{LG}_{\ell,p}$ in the main text corresponds to $\mathrm{LG}^{3\mathrm{D}}_{\ell,p}(R,\phi,z;w)$ for some fixed value of w (and of course k).

Although the equation (S2.2) is more useful for calculations, the form (S2.3), in which complex factors have had their modulus and phase separated, is easier to interpret physically. In particular, the LG beam has two important z-dependent phase factors: the Gouy phase $\mathrm{e}^{\mathrm{i}(|\ell|+2p+1)\arctan(z/z_\mathrm{R})}$, and the wavefront curvature $\mathrm{e}^{\mathrm{i}\,R^2 z z_\mathrm{R}/w^2(z_\mathrm{R}^2+z^2)}$. Neither of these contribute in the waist plane $z = 0$.

For each value of z (i.e. transverse plane), the LG modes are an orthonormal basis for 2D complex amplitude distributions. The simplest such plane is the focal plane $z = 0$, for which

$$\mathrm{LG}^{2\mathrm{D}}_{\ell,p}(R,\phi;w) \equiv \mathrm{LG}^{3\mathrm{D}}_{\ell,p}(R,\phi,z=0;w) = \sqrt{\frac{p!}{\pi(|\ell|+p)!}}\frac{(R/w)^{|\ell|}\mathrm{e}^{\mathrm{i}\ell\phi}}{w}\mathrm{L}^{|\ell|}_p\left(\frac{R^2}{w^2}\right)\exp\left(-\frac{R^2}{2w^2}\right). \tag{S2.4}$$

As the fields are synthesised by 2D holograms in a beam's transverse plane, we approach the 3D field design primarily in terms of the field distributions in the 2D field [17]. Following standard linear space theory, any scalar solution $\psi(R,\phi,z)$ of the paraxial wave equation can be written as a superposition

$$\psi(R,\phi,z) = \sum_{\ell,p} c_{\ell,p}\mathrm{LG}^{3\mathrm{D}}_{\ell,p}(R,\phi,z;w), \tag{S2.5}$$

where the generally complex coefficients are found from the 2D inner product integral

$$c_{\ell,p} = \int_0^\infty\int_0^{2\pi}\psi(R,\phi,z=0)\mathrm{LG}^{2\mathrm{D}*}_{\ell,p}(R,\phi;w)R\,\mathrm{d}\phi\,\mathrm{d}R. \tag{S2.6}$$

2.2. Mapping from 3D real space to the optical hypersphere

Any transverse optical field defines a mapping from 3D real space, parameterised by cartesian x, y, z or cylindrical polar R, ϕ, z, to the optical hypersphere, which is parameterised in terms of X, Y, Z, ω, θ, φ, or α, β, γ as discussed in Sec. 1.2, each of which are different ways of expressing the complex components $E_\mathrm{R} = U, E_\mathrm{L} = V$. In this section we will be careful distinguish the coordinates in the target space (i.e. the optical hypersphere), and the topological mapping to them from real space, as the notation of the complex fields $E_\mathrm{R}(x,y,z)$ and $E_\mathrm{L}(x,y,z)$ suggests both.

The most direct such map would be an optical field which maps each point of real space to the corresponding point in the stereographic projection of the optical hypersphere, i.e. $(x,y,z) \longrightarrow (X,Y,Z)$. Under such a map, each point of real space would correspond to a unique optical state (including the limiting point at infinity), wrapping around the optical hypersphere exactly once. However, such a mapping is not compatible with a physically realisable optical field, satisfying the paraxial equation.

As a step towards this, we define the following real-space counterparts to U and V of equation (S1.17)

$$u(x,y,z) = \frac{x^2+y^2+z^2-1+2\,\mathrm{i}\,z}{x^2+y^2+z^2+1} = \frac{R^2+z^2-1+2\,\mathrm{i}\,z}{R^2+z^2+1}, \qquad v(x,y,z) = \frac{2(x+\mathrm{i}\,y)}{x^2+y^2+z^2+1} = \frac{2R\,\mathrm{e}^{\mathrm{i}\phi}}{R^2+z^2+1}. \tag{S2.7}$$

In the correspondence between x, y, z and X, Y, Z, $\mathrm{Re}\, u$ corresponds to $-\,\mathrm{Im}\, U$, as we explain below.

If we could realise a field with $E_{\mathrm{R}} = u(x, y, z)$ and $E_{\mathrm{L}} = v(x, y, z)$, we would realise a reflection of the skyrmionic hopfion (reflected in the $n_W = 0$ plane), but as stated above, this is not possible physically with solutions of the paraxial wave equation. The vortex function $\psi_1(x, y, z) = 2(x \pm \mathrm{i}\, y)$, formally satisfies the paraxial equation, and can be taken as a local expansion of a physically realisable ψ_{L} giving the right form agreeing with the numerator of v. Furthermore, the function $\psi_2(x, y, z) = x^2 + y^2 - 1 + 2\,\mathrm{i}\, z/k$ also satisfies the paraxial wave equation, so can represent a quadratic expansion of a physical field [18]. It has a circular vortex line around the axis in the $z = 0$ plane, and in fact agrees with the numerator of u when z is small.

The field ψ_1 satisfies the paraxial wave equation, although its conjugate ψ^* does not; u was defined in equation (S2.7) to approximately agree with ψ_1, in spite of it mapping to a reflection of the hypersphere. Positive orientation can be restored to our mapping by choosing to generate ψ_2^* in the other field, rather than ψ_2, i.e. a beam with a negative, rather than positive, axial vortex. This guarantees that the covering of the hypersphere by the real space beam has a positive sense, and the net mapping degree—the experimental Skyrme number—should be close to $+1$.

Therefore beams with $\psi_{\mathrm{L}} = \psi_1^*$ and $\psi_{\mathrm{R}} = \psi_2$, realise the correct topological mapping from real space to the hypersphere. Although not perfect realisations of the $1-1$ mapping between the two 3D spaces, the physically realisable optical field we will describe is a smooth deformation of these, homotopically equivalent to the Π_3 mapping around the hypersphere, except a small neighbourhood near ∞ as we truncate within a finite experimental volume.

2.3. Topological design via C line skeleton

We thus design the structured fields $E_{\mathrm{R}}(x, y, z)$ and $E_{\mathrm{L}}(x, y, z)$ by matching the topology of $\psi_{\mathrm{R}}(x, y, z)$ and $\psi_{\mathrm{L}}(x, y, z)$ to the complex fields $\psi_2(x, y, z)$ and $\psi_1^*(x, y, z)$, from Sec. 2.2 as closely as possible in the focal volume. The main design principle for the topology of the skyrmionic hopfion is therefore a vortex loop around the beam axis in the RH component (which corresponds to a LH C line loop in the full field), threaded by a straight vortex line along the z-axis in the LH component (corresponding to a RH C line).

As with other topological singular configurations designed in 3D [17, 19, 20], each of the RH and LH components is considered as a superposition of LG modes. The simplest way to generate a vortex ring in ψ_{R} is in the focal plane, by a superposition of $\mathrm{LG}_{0,0}$ and $\mathrm{LG}_{0,1}$. Thus, for this component all coefficients (S2.6) are zero except $c_{0,0}$ and $c_{0,1}$, which we redefine them in terms of new parameters a, b as:

$$c_{0,0} = -a + b, \qquad\qquad c_{01} = -b. \tag{S2.8}$$

Therefore, the RH field is

$$\psi_{\mathrm{R}} = \frac{\mathrm{e}^{-R^2/2w^2(1+\mathrm{i}\, z/z_{\mathrm{R}})}}{\sqrt{\pi} w (1 + \mathrm{i}\, z/z_{\mathrm{R}})^3} \left[b(R^2/w^2 + 2\,\mathrm{i}\, z/z_{\mathrm{R}}(1 + \mathrm{i}\, z/z_{\mathrm{R}}) - a(1 + \mathrm{i}\, z/z_{\mathrm{R}})^2 \right]. \tag{S2.9}$$

Here, the coefficients a and b tailor the radius of the vortex ring, the shape of the vortex core, and the related intensity. The superposition is chosen so the optical vortex ring has radius $R = R_0 = \sqrt{a/b} w$ $(a, b > 0)$ in the focal plane $z = 0$. In order to get a positive Skyrme number, it is necessary to use a negatively signed axial vortex in ψ_{L}. This is equivalent to matching the topology of $v^*(x, y, z)$, necessary to guarantee the mapping preserves the sign of 3D volume is preserved; more details are in Supplementary Note 4. For ψ_{L}, therefore, all $c_{\ell,p}$ are zero except for $c_{-1,0}$, which we choose to be

$$c_{-1,0} = 2c, \tag{S2.10}$$

involving the new constant $c > 0$. Thus, the LH component is given by

$$\psi_{\mathrm{L}} = \frac{\mathrm{e}^{-R^2/2w^2(1+\mathrm{i}\, z/z_{\mathrm{R}})}}{\sqrt{\pi} w (1 + \mathrm{i}\, z/z_{\mathrm{R}})^3} \left[2cR/w\, \mathrm{e}^{-\mathrm{i}\phi}(1 + \mathrm{i}\, z/z_{\mathrm{R}}) \right]. \tag{S2.11}$$

This expression outside the square brackets has the same nonzero prefactor as the expression for ψ_{R}. From the perspective of the polarization, all that matters are the parts in the square brackets of equations (S2.9) and (S2.11).

Given fixed values of k and w, the optical skyrmionic hopfion forms in a parameter range of a, b, c. The criteria by which the parameters are optimised for experiments are reported in the Methods. When $a = b = c = 1$, the terms in the bracket become $R^2/w^2 - 1$ and $2R/w\, \mathrm{e}^{-\mathrm{i}\phi}$ and correspond (up to scaling), respectively, to the numerators of the

complex 3-sphere coordinate fields u and v^* from equation (S2.7) [21]. However, the z-dependence of the propagating light fields is different from the full u, v (v^*) fields. This is similar to the design of optical vortex knots [17], whose design is based on more general complex scalar fields (e.g. Milnor polynomials) which have the same amplitude in the focal plane and same global 3D topology, although the functional form is different.

Supplementary Note 3: Optical generation and detection details

In this note we give an overview about the digital propagation method that is used to generate transverse planes at different propagation distances around the focus on the camera, the phase and polarization measurement technique and discuss the error rate on basis of their possible origins in the setup.

3.1. Measuring the optical skyrmionic hopfion

Digital propagation is an approach that allows for the investigation of different z-planes of a 3D structured paraxial light field without mechanically moving the observation plane. The field of interest itself is propagated by varying the Fourier hologram on a SLM [22, 23] as follows. In terms of the Fourier representation, the scalar 3D light field with transverse position $\boldsymbol{r}_\perp$ physically propagating in the $+z$-direction can be represented by $\psi(\boldsymbol{r}_\perp, z) = \mathcal{F}^{-1}(\mathcal{F}(\psi(\boldsymbol{r}_\perp, 0))\mathrm{e}^{\mathrm{i}k_z z})$ with $\mathcal{F}^{-1}$ denoting the inverse Fourier transform and, in the paraxial approximation, $k_z \approx k - (k_x^2 + k_y^2)/(2k)$.
Since we display a Fourier hologram $\mathcal{F}(\psi(\boldsymbol{r}_\perp, 0))$ per scalar mode on the SLM for generating the skyrmionic hopfion structure in the focus of L3 (see Supplementary Fig. 4b), we are able to digitally propagate the differently polarized scalar fields $\psi(\boldsymbol{r}_\perp, z)$ and, thereby, the total topological texture in the z-direction, by multiplying the Fourier holograms at different transverse planes with the according phase factor $\mathrm{e}^{\mathrm{i}k_z z}$. For our analysis of the skyrmionic hopfion structure, we propagate in the range of z (see Methods) in 100 steps of 100λ each, for investigating the phase as well as polarization per z-plane.

For the polarization analysis per transverse plane, a rotating QWP and fixed horizontally aligned polarizer (Pol) are mounted in front of a camera (see Supplementary Fig. 4c). Depending on the rotation angle Θ of the QWP, the camera records spatially resolved intensity patterns described by $I(\Theta) = \frac{1}{2}(s_0 + s_1 \cos^2(2\Theta) + s_2 \cos(2\Theta)\sin(2\Theta) + s_3 \sin(2\Theta))$ [24], where s_0, s_1, s_2, s_3 are the unnormalized Stokes parameters, s_0 is total intensity, $s_j = s_0 S_j$, for $j = 1, 2, 3$. To suppress time-dependent errors, every intensity profile is the arithmetic mean of 10 intensity patterns. The Stokes parameters are measured recording the intensity patterns within the transverse plane of interest for 15 different angles Θ. The resolution of the measurement is given by the camera (1280 x 1024 px), therefore the transverse polarization profile is determined across a single plane [25, 26]. To use the full resolution of the camera, the beam structure is magnified by lens L4 with a factor of 16 onto the camera. The combination of polarimetry across transverse planes with digital propagation (see above) allows the full detection of the 3D polarization texture in the focal volume.

In order to retrieve not only the polarization but also the phase information from the realised 3D vectorial light field, in addition we implement digital holographic phase measurements [27]. This method allows for the determination of the transverse phase structure in a fixed z-plane of one orthogonal component (scalar field) of the beam relative to a reference beam. For phase metrology, we coherently interfere the scalar beam (signal beam), $\psi(\boldsymbol{r}_\perp, z)$, off-axis with a reference beam, $\psi_{\mathrm{ref}}(\boldsymbol{r}_\perp) = |\psi_{\mathrm{ref}}|\mathrm{e}^{\mathrm{i}k_R R}$ (plane wave). Both beams are of the same circular polarization. The reference beam illuminates the camera at an angle Φ to the signal beam (see Supplementary Fig. 4d), generating an interference pattern whose intensity is $I(\boldsymbol{r}_\perp) = |\psi(\boldsymbol{r}_\perp) + \psi_{\mathrm{ref}}(\boldsymbol{r}_\perp)|^2$. The mixed term $|\psi||\psi_{\mathrm{ref}}|\mathrm{e}^{\mathrm{i}(\phi - k_R R)}$ embedded in this interference pattern contains the desired phase information $\chi(\boldsymbol{r}_\perp)$. This term is extracted by a suited low-pass filter located at k_R, the k-vector of the reference beam. By centering and performing an inverse Fourier transform of this cropped pattern we receive the pure phase profile of the signal beam. Similar to polarimetry, the beam is magnified and the intensity measurements are averaged.

3.2. Errors in measurements

In general, various systematic errors might occur within the experiment. For instance, these may include time dependent fluctuations within the system for the generation and measurement of the skyrmionic hopfion structure, and constant inaccuracies of optical components. We observe imperfections of the beam's intensity profile even before the beam is

reflected on the SLM. These imperfections are from the laser source itself and the optical components that guide the beam to the SLM. To address this issue, a beam cleaning is installed immediately before the SLM to provide the best possible profile. During its way through the setup, the beam profile is affected by polarizing optics (wave plates and polarizer) and imperfections in the SLM. We minimize these systematic errors by precise adjustment, implementation of high-quality optical components, as well as phase profile corrections [28].

Within the measurement procedure of our VFFR method, minor deviation per pixel for polarization and phase measurements can be observed. The average error per pixel within the transverse plane is experimentally determined to be $< 4\%$ and $< 2\%$ for polarization and phase measurements, respectively. Since we use the arithmetic mean of ten measurements for each picture, this errors represent the standard deviation within ten measurements at the same $(\boldsymbol{r}_\perp, z)$-position within the beam. Note that this average error is calculated from the errors per pixel, which vary spatially. As representative examples, Supplementary Fig. 7 shows a density plot at the focal plane ($z = 0$) of the deviation of the polarization component S_3 of the skyrmionic hopfion structure (a) and the phase χ_L (b).

Errors particularly affect the polarization measurements around the nodal line of E_R (i.e. the LH C line loop). The superposition of E_R and E_L in this area is very sensitive to small fluctuations in the intensity and phase of one of the beams. Slight fluctuations cause rotations of the polarization profile. However, observed deviations in polarization do not hinder the formation of the topology. On the other hand, the phase structure reveals a larger error close to the optical axis, around the phase singularity in E_L (i.e. the axial RH C line). This effect is due to the higher phase gradient in this area, which causes small spatial vibrations in the experimental setup to have a significant effect on the phase variations between repetitive measurements. In spite of this, the phase singularity propagates close to the optical axis, as visible in Supplementary Fig. 6. Thus, it is in a good agreement with the theoretical prediction.

Any errors here are inherited in the determination of the experimental Skyrme number (see Methods). Despite visual deviations between the experimental and the theoretical structure in light and described errors, we were able to realise an optical skyrmionic hopfion with a small deviation of Skyrme number to the theoretically determined value (see Sec. 5.2).

Supplementary Note 4: Skyrme density, helicity and optical currents

Here we describe in more detail the Skyrme density, the continuous topological charge density which represents the covering of the optical hypersphere by the position-dependent phase and polarization in the optical field volume. Starting with its mathematical definition as the jacobian of the transformation between 3D configuration space and the optical hypersphere, we then put it into a more physical context related to singular optics and topological helicity. This extends to a discussion of the chosen volume over which the Skyrme number is calculated, and then further theoretical features of the model.

4.1. Skyrme density as the jacobian of the mapping from real space to optical hypersphere

We describe the basic derivation of the Skyrme density as the jacobian of the topological Π_3 mapping from the experimental real space volume to the optical hypersphere. This is motivated by the analogous 2-dimensional mapping to the Poincaré sphere.

4.1.1. Baby polarization skyrmion density and covering the Poincaré sphere

The Poincaré sphere is parameterised by azimuth angle α, $-\pi < \alpha \leq \pi$, and polar angle β, $0 \leq \beta \leq \pi$ (defined in equations (S1.10) and (S1.11)). Its area element is, as usual, $\sin\beta\, \mathrm{d}\alpha\, \mathrm{d}\beta = \mathrm{d}\alpha\, \mathrm{d}(\cos\beta)$.

As a 2-surface, it has a standard east-north or south-east orientation. This corresponds to the sense of increase of azimuth α in the northern hemisphere, and its opposite in the southern, as discussed in Sec. 1.2. This means that a mapping from the x, y-plane to the Poincaré sphere acquires a jacobian factor, which we call the 2D Skyrme density $\Sigma^{(2D)}$,

$$\Sigma^{(2D)}\, \mathrm{d}x\, \mathrm{d}y = \frac{1}{4\pi}\, \mathrm{d}\alpha\, \mathrm{d}(\cos\beta), \tag{S4.1}$$

where a normalizing factor of the area of the 2-sphere has been included on the right-hand side. Therefore, including the orientation, $\Sigma^{(2D)} = \partial_x\alpha\partial_y(\cos\beta) - \partial_y\alpha\partial_x(\cos\beta) = \hat{\mathbf{z}} \cdot \nabla(\alpha) \times \nabla(\cos\beta)$. From the second equality, we define the baby

skyrmion density vector

$$\Sigma^{\mathrm{b}} = \nabla\alpha \times \nabla(\cos\beta). \tag{S4.2}$$

Thus $\Sigma^{(2\mathrm{D})} = \Sigma^{\mathrm{b}}_z$, and in general the density of covering the Poincaré sphere in any plane in 3D, with normal vector $\widehat{\boldsymbol{u}}$, can be found using $\widehat{\mathbf{u}} \cdot \Sigma^{\mathrm{b}}$. This can be compared to the continuous 2D topological charge density in full Poincaré-type beams [29–31] and baby skyrmions in other systems [32–38].

Full Poincaré beams are propagating beams with position-dependent polarization such that the integral of Σ^{b}_z in each transverse plane integrates to an integer; this is guaranteed when, as $x^2 + y^2 \to \infty$, the polarization state tends to a constant independent of direction. This effectively allows point compactification of the polarization states in the plane, and thus a topological mapping from 2D real plane to 2-sphere [39].

4.1.2. Skyrme density and covering the optical hypersphere

A skyrmionic hopfion is a mapping from 3D real space to the optical hypersphere, where the position-dependent optical polarization and phase parameters wrap around the hypersphere. We can find the corresponding 3D Skyrme density by analogy with the 2D case.

We therefore are considering a mapping from 3D real space with cartesian coordinates x, y, z to the optical hypersphere with polarization parameters α, β and phase parameter γ (defined in equation (S1.13)). We discussed previously, in Sec. 1.2, that the 3-sphere of unit radius has total volume $2\pi^2$, and as derived in equation (S1.24), the normalized volume element of the 3D hypersphere is $(2\pi^2)^{-1}\,\mathrm{d}^3\Omega = (16\pi^2)^{-1}\,\mathrm{d}\alpha\,\mathrm{d}(\cos\beta)\,\mathrm{d}\gamma$, with positive volume orientation given by $(\alpha, \cos\beta, \gamma)$. Therefore, by analogy with equation (S4.1), the 3D Skyrme density Σ is the jacobian

$$\Sigma\,\mathrm{d}x\,\mathrm{d}y\,\mathrm{d}z = \frac{1}{16\pi^2}\,\mathrm{d}\alpha\,\mathrm{d}(\cos\beta)\,\mathrm{d}\gamma, \tag{S4.3}$$

where again the right hand side is normalized so the integral over the whole 3-sphere gives unity.

It would also be possible to use 3-sphere polar angles φ, θ, ω as in equation (S1.18) to define the Skyrme density, but the polarization parameters α, β, γ are physically more natural to describe the optical state. These latter parameters are those of the Hopf fibration of the 3-sphere: a small interval $\delta(\cos\beta) = \delta S_3$ (corresponding to a thin 'vertical' slice of the Poincaré sphere) corresponds to a thickened torus in the 3-sphere, on which curves of fixed γ and fixed α wind in opposite directions as Hopf circles.

In a skyrmionic hopfion, the α, β, γ parameters wind around their angles in a sense preserving overall positive orientation, guaranteeing the integral of Σ over 3D real space entirely covers the optical hypersphere an integer number of times, defining the Skyrme number.

This full 3D skyrmion is similar to topological textures in other systems. A Shankar monopole, for instance, is a texture in fields characterised by a 3D orthogonal frame, i.e. an orthogonal 3×3 matrix [40–42]. The space of such matrices, the Lie group SO(3), is doubly covered by the 3-sphere; in terms of our parameters, γ closes after a 2π cycle, not 4π.

Other textures of interest are pure hopfions, characterised by mappings from 3D real space to the 2D sphere (such as fields of unit vectors representing magnets, or directors representing liquid crystal orientations). Their relation with our optical Skyrme density is discussed in the next section.

4.2. Polarization hopfions and optical currents

A characteristic feature of any topological texture of closed filaments filling 3D space is that they are mutually linked – inherited by the map between real space and the 3-sphere parameterised by the Hopf fibration which has this property. For polarization textures we can consider this in the context of topological features usually considered in singular optics such as optical vortices and C lines.

Optical vortices are zeros of a complex scalar field ψ representing a polarized scalar component. Mathematically, the points $\mathbf{r}$ satisfying $\psi(\mathbf{r}) = 0$, that is, the preimages of 0. These correspond to the set of vortex lines, which can be infinite lines, loops, or knots [16]. In fact, any complex constant ψ_0 has a preimage defined by $\psi(\mathbf{r}) = \psi_0$, and, just like vortex lines, these are typically filaments in 3D.

A similar argument holds for polarization filaments, where now the complex scalar is $\Psi = E_{\mathrm{L}}/E_{\mathrm{R}} = \psi_{\mathrm{L}}/\psi_{\mathrm{R}}$ as defined in equation (S1.14), whose argument is α and modulus is a monotonic function of β and S_3. However, on LH C lines where $E_{\mathrm{R}} = 0$, Ψ may take the value ∞. (This notion may be made more mathematically rigorous with the notion of sections

of complex bundles, which we avoid to remain close to standard terminology in optics.) As discussed in Supplementary Note 1, this means the parameter space of the map corresponds topologically to a sphere, allowing Hopf textures and, with phase, 3D skyrmions.

The tangent to the filament at each point is given by the vorticity field

$$\boldsymbol{\omega} = \frac{-\mathrm{i}}{2} \frac{\nabla\Psi^* \times \nabla\Psi}{(1+\Psi^*\Psi)^2}. \tag{S4.4}$$

This is similar to the vorticity field $\boldsymbol{\omega}^{\mathrm{sc}} \equiv -\mathrm{i}\,\nabla\psi^* \times \nabla\psi$ for a normal complex scalar field ψ [17], but here with an extra denominator regularising the divergence in the neighbourhood of $\Psi = \infty$. Such a field is the curl of an optical current associated with the scalar Ψ,

$$\mathbf{J} = \frac{-\mathrm{i}}{4\pi} \frac{\Psi^*\nabla\Psi - \Psi\nabla\Psi^*}{1+\Psi^*\Psi}, \tag{S4.5}$$

and evidently $\boldsymbol{\omega} = \nabla \times \mathbf{J}$. Again, apart from the regularising denominator, this is the standard current associated with a complex scalar field, $\mathbf{J}^{\mathrm{sc}} = \mathrm{Im}\,\psi^*\nabla\psi$.

In the theory of knotted fields, the linking of field lines of divergence-free vector fields are often of interest; we write such a field as a magnetic field $\mathbf{B}$ (although we are interested here in $\boldsymbol{\omega}$). The continuous linking density of the field lines of $\mathbf{B}$ is given by the helicity density $\mathbf{B}\cdot\mathbf{A} = \mathbf{A}\cdot\nabla\times\mathbf{A}$ for vector potential $\mathbf{A}$ [38, 43–46]. The integral over all 3D real space gives the total helicity of the field, for a magnetic field related to duality symmetry, and for polarization textures with tangents specified by $\boldsymbol{\omega}$, related to the global linking of polarization filaments.

However, it is clear from equation (S4.5) that the corresponding polarization helicity density $\mathbf{J}\cdot\boldsymbol{\omega} = 0$ everywhere except where $\Psi = \infty$, which has not been suitably regularised. An approach to this was considered in [47] (following from similar calculations in [48]), where the singularity on the LH C line in $\mathbf{J}$ is resolved by a singular gauge transformation

$$\mathbf{J} \;\rightarrow\; \mathbf{J} + \frac{-\mathrm{i}}{4\pi}\nabla\log\frac{E_{\mathrm{R}}}{E_{\mathrm{R}}^*} \;\equiv\; \widetilde{\mathbf{J}}. \tag{S4.6}$$

This new current $\widetilde{\mathbf{J}}$ again satisfies $\nabla\times\widetilde{\mathbf{J}} = \boldsymbol{\omega}$, but now the helicity is nonzero. Using equation (S4.6), the new current is

$$\widetilde{\mathbf{J}} \;=\; \frac{1}{2\pi}\mathrm{Im}(E_{\mathrm{R}}^*\nabla E_{\mathrm{R}} + E_{\mathrm{L}}^*\nabla E_{\mathrm{L}}) \;=\; \frac{1}{2\pi}\frac{\mathrm{Im}(\psi_{\mathrm{R}}^*\nabla\psi_{\mathrm{R}} + \psi_{\mathrm{L}}^*\nabla\psi_{\mathrm{L}})}{(|\psi_{\mathrm{R}}^2 + |\psi_{\mathrm{L}}^2|)}, \tag{S4.7}$$

that is, the regularised current $\widetilde{\mathbf{J}} = \mathbf{J}_{\mathrm{o}}$, the orbital current associated with the field; the numerator is the sum of the two scalar currents associated with the two field components, appropriately normalized. Added to the spin current (which does not play a significant role in the present situation), this gives the Poynting vector of the paraxial optical field [49].

How is this related to the Skyrme density of the previous part? In terms of the angle parameters α, β, γ, it is easy to see that $\mathrm{Im}\,E_{\mathrm{R}}^*\nabla E_{\mathrm{R}} = \frac{1}{4}(1+\cos\beta)(\nabla\gamma - \nabla\alpha)$ and $\mathrm{Im}\,E_{\mathrm{L}}^*\nabla E_{\mathrm{L}} = \frac{1}{4}(1-\cos\beta)(\nabla\gamma + \nabla\alpha)$, implying that

$$\mathbf{J}_{\mathrm{o}} \;=\; \tfrac{1}{4\pi}\left(\nabla\gamma - \cos\beta\nabla\alpha\right), \tag{S4.8}$$

and

$$\boldsymbol{\omega} \;=\; \nabla\times\mathbf{J}_{\mathrm{o}} \;=\; \tfrac{1}{4\pi}\nabla\alpha\times\nabla(\cos\beta) \;=\; \Sigma^{\mathrm{b}}, \tag{S4.9}$$

that is, the vorticity of the polarization field is the baby skyrmion density vector (see equation (S4.2)). This is elegant but is not surprising, as the vorticity vector arises as the jacobian between real space and field space when counting (discrete) topological charge densities in singular optics [3, 50]. As discussed in the main text, the topological role of helicity in flows has a similar expression in terms of an appropriate velocity field [32, 45, 47, 51–53].

We can summarise the preceding discussion in terms of Skyrme density as follows. The directions of the polarization filaments are given by a vector field determined by $\boldsymbol{\omega} = \Sigma^{\mathrm{b}}$. The natural vector potential associated with this divergence-free field is singular at the singularities of α (i.e. the C lines), but when regularised by adding the phase gradient $\nabla\gamma$, the polarization helicity density reduces to the 3D Skyrme density, as indeed it must:

$$\Sigma \;=\; \nabla\gamma\cdot\Sigma^{\mathrm{b}} \;=\; \mathbf{J}_{\mathrm{o}}\cdot\boldsymbol{\omega}, \tag{S4.10}$$

also equivalent to equation (4) in the main text.

The preceding discussion also illustrates that further gauge transformations—that is, adding nonsingular scalar fields to γ—does not change the overall Skyrme number. In the study of pure hopfions (i.e. maps from 3D real space to a 2-sphere), whose filament directions are given by **B** but there is no natural phase, it is usually necessary to construct a 'vector potential' to get the correct helicity density [44, 46].

Similarly, if we choose to focus purely on optical polarization hopfions, we can choose an appropriate phase. The associated helicity equation (S4.10) integrated over all 3D space characterises the interlinking of polarization filaments, and globally the number of times the 3D field wraps around the Poincaré sphere. Any convenient choice of phase function (which is appropriately singular on C lines) suffices for this choice. Several choices are discussed in the following section, which applies the preceding analysis to our model field, as introduced in the Methods and Supplementary Note 2.

We also remark on textures for a normal (non-infinity) complex scalar field ψ. Assuming that ψ, as a regular complex-valued function, is nowhere infinite (not even at spatial infinity) means the total set of complex values the field takes is of finite range for a finite set, and hence the texture of its filaments (labelled by ψ_0) cannot be topological in spite of some filaments being knotted and linked. If we choose to regarding the vortex lines of ψ (i.e. preimages of zero) being special, we can choose the phase gradient $\nabla \arg \psi$, analogous to the superfluid velocity, as the appropriate current vector. The curl of $\nabla \arg \psi$ vanishes everywhere apart from δ-concentrations on the vortex line; careful regularisation gives a weighting to the screw dislocation nature of the wave vortex [50]. As the global helicity of the field must be zero, the phase around a vortex loop must unwind (i.e. self-link) whenever it is threaded (i.e. linked) with another vortex line [54].

4.3. Integrating the Skyrme and Hopf densities of the model of skyrmionic hopfion

The topology of the optical skyrmionic hopfion resides in the map from 3D configuration space to the optical hypersphere, parameterised by α, β, γ by the fields (S2.9), (S2.11) with the numerical values of the coefficients given in the Methods. As discussed in the main text in the context of the experiment, the polarization of these fields consists of a texture of filaments of constant polarization resembling the Hopf fibration (main text Fig. 3), and phase varying along them establishing a Π_3 mapping between the two 3D spaces. The Skyrme density is given by $\Sigma = \frac{1}{16\pi^2}\nabla\gamma \cdot (\nabla\alpha \times \nabla(\cos\beta))$.

As described in the Methods, the coefficients of this model field have been chosen optimally to concentrate the intensity and Skyrme density. Supplementary Fig. 8a shows a cross-section of the Skyrme density for this field (equivalent to the 3D plot in the inset to Fig. 4c in the main text). The Skyrme density for the model field is strongly concentrated around the C line ring, with most of the contours shown in the plot being several orders of magnitude smaller than the maximum density.

Provided the neighbourhood of the vortex ring is included in the integration volume, we expect the integrated Skyrme density to be close to 1. Supplementary Fig. 8b demonstrates the result of this integral as a function of the transverse ($L_\perp$) and longitudinal ($L_\parallel$) dimensions of the integration volume given by $|x| \leq L_\perp$, $|y| \leq L_\perp$, $|z| \leq L_\parallel$. Most of the area shows 99% of the 3-sphere volume is filled. This justifies our claim that the optical skyrmonic hopfion is particle-like – the integration of the topological charge density is independent of the size of the sufficiently large integration box, and is very close to unity.

The evaluation of the measured Skyrme number from the experimental data is described in Methods and Supplementary Note 5. As explained there, rather than construct a smooth interpolation of Σ from the data then integrate numerically, we calculate the fraction of the 3-sphere filled by a piecewise linear map constructed directly from the data. Even with high-performance computers this approach requires computational effort. Justified by the particle-like nature of the skyrmionic hopfion, we limit the calculation of the experimental Skyrme number to the cuboid of transverse size $L_\perp = 1.84w = 99.4\,\mu\text{m}$ and longitudinal size $L_\parallel = 0.768z_\text{R} = 26.6\,\text{mm}$ indicated in Supplementary Fig. 8, a and b, in cyan. The numerical integral of the model field in this restricted cuboid is 0.9966, i.e. 99.7% of the 3-sphere volume. For reference, the total measured volume (see Methods) is the cuboid given by $x_\text{max} = 3.13w = 170\,\mu\text{m}$, $y_\text{max} = 3.91w = 212\,\mu\text{m}$ and $z_\text{max} = 0.768z_\text{R} = 26.6\text{mm}$. The numerical integral of Σ for the model field in the measured volume is 0.997 and is also indicated in Supplementary Fig. 8b in cyan. Thus, to 3 significant figures, the Skyrme number in the reduced volume is no different from the integrated density in the total measured volume.

We note that there is a contour in Supplementary Fig. 8b for $L_\perp > 2w$, $L_\parallel > 1.5z_\text{R}$ where the integrated Skyrme volume equals 1. This due to the properties of gaussian beams used to realise the field, which is described, with a mathematical analysis of the asymptotic value of the field for large R and z, in the next subsection. However, for practical purposes it is challenging to measure the field's polarization and phase so far from the focal point, as the intensity is small and subject to experimental imperfections in this region.

4.4. Theoretical considerations of model skyrmionic hopfion

We now consider some of the topological details associated with our model field (S2.9), (S2.11) in more detail. For convenience, we use dimensionless cylindrical coordinates $\overline{R} = R/w$, $\overline{z} = z/z_{\mathrm{R}}$, but keeping the coefficients a, b, c. To simplify the expressions, we write $1 + \overline{z}^2 = W$.

The Skyrme density $\Sigma = \frac{1}{16\pi^2}\nabla\gamma \cdot (\nabla\alpha \times \nabla(\cos\beta))$ for the superposition of LG modes given by equations (S2.9) and (S2.11), in terms of these dimensionless coordinates, is

$$\begin{aligned}\Sigma \;=\; &\Big(c^2(a^2(2-\overline{R}^2)\overline{z}W^3 + b^2(\overline{R}^6\overline{z}W + 16\overline{z}^2\overline{z}W^2 - 2\overline{R}^4(3-\overline{z}^2) + 4\overline{R}^2(1-3\overline{z}^2-5\overline{z}^4-\overline{z}^6))\\ &-4ab(2\overline{R}^4\overline{z}^2 - \overline{z}W^2(1-3\overline{z}^2) + \overline{R}^2(1-3\overline{z}^2-5\overline{z}^4-\overline{z}^6)))\Big)\\ &/\pi^2\overline{z}W(4c^2\overline{R}^2\overline{z}W + a^2\overline{z}W^2 + b^2(4\overline{z}^2 + (\overline{R}^2-2\overline{z}^2)^2) - 2ab(\overline{R}^2(1-\overline{z}^2) + 2\overline{z}^2\overline{z}W))^2. \end{aligned} \qquad \text{(S4.11)}$$

For $\overline{R} \gg 1$, we have that $\Sigma \sim c^2/\pi^2 b^2 \overline{R}^2 + O(1/R^4)$. Therefore the integral over infinite radius diverges logarithmically. We will consider the meaning of this in the following.

4.4.1. Polarization parameters

From equation (S1.10), the gradient $\nabla\alpha$ for the model field in cylindrical coordinates is

$$\nabla\alpha \;=\; -\frac{\widehat{\boldsymbol{\phi}}}{\overline{R}} + \frac{\left[4b(b-a)\overline{R}\overline{z}(1+\overline{z}^2)\widehat{\mathbf{R}} + 2ab(1+2\overline{z}^2(1+\overline{R}^2) + \overline{z}^4 - a^2(1+\overline{z}^2)^2 - b^2\overline{R}^2(2+6\overline{z}^2-\overline{R}^2)\widehat{\mathbf{z}}\right]}{(1+\overline{z}^2)((a-b\overline{R}^2)^2 + 2\overline{z}^2(a^2-ab(2-\overline{R}^2)+2b^2(1-\overline{R}^2)) + (a-2b)^2\overline{z}^4)}, \qquad \text{(S4.12)}$$

which is singular along and circulates around the C lines: along the axis $\overline{R} = 0$ and the circle $(\overline{R}, \overline{Z}) = (\sqrt{a/b}, 0)$.

From (S1.11), $\cos\beta = S_3$, which is given by

$$S_3 = 1 - \frac{8c\overline{R}^2(1+Z^2)}{4c^2\overline{R}^2\overline{z}W + a^2\overline{z}W^2 + b^2(4\overline{z}^2 + (\overline{R}^2-2\overline{z}^2)^2) - 2ab(\overline{R}^2(1-Z^2) + 2\overline{z}^2\overline{z}W)}. \qquad \text{(S4.13)}$$

Its gradient $\nabla(\cos\beta)$ is

$$\frac{16c^2\overline{R}\left(\overline{z}W(b\overline{R}^4 - \overline{z}W(a^2+(a-2b)^2\overline{z}^2))\widehat{\mathbf{R}} + \overline{R}\overline{z}(a^2\overline{z}W^2 + 4ab(\overline{R}^2 - \overline{z}W^2) + b^2(4\overline{z}W^2 - \overline{R}^2(4+\overline{R}^2))\widehat{\mathbf{z}}\right)}{\left(4c^2\overline{R}^2\overline{z}W + a^2\overline{z}W^2 + b^2(4\overline{z}^2 + (\overline{R}^2-2\overline{z}^2)^2) - 2ab(\overline{R}^2(1-\overline{z}^2) + 2\overline{z}^2\overline{z}W\right)^2}. \qquad \text{(S4.14)}$$

Together, these determine the polarization behavior of the skyrmionic hopfion, and the field's baby skyrmion/full Poincaré beam nature in any plane.

We now consider the Skyrme density and Hopf density (polarization helicity) of the model field for ψ_{R} and ψ_{L} given in equation (S2.9) and equation (S2.11) respectively, found as superpositions of LG modes.

4.4.2. 2D baby Skyrme density/full Poincaré beam nature

The model beam's baby Skyrme density (polarization vorticity) vector is, in cylindrical coordinates,

$$\begin{aligned}\Sigma^{\mathrm{b}} = &\frac{4b^2}{\pi((1+\overline{Z}^2)(4b^2\overline{R}^2 + a^2W - 4ab\overline{z}^2) + b^2((\overline{R}^2-2\overline{z}^2)^2 + 4\overline{z}^2) - 2ab\overline{R}^2(1-\overline{z}^2)^2}\\ &\times\Big[-b\overline{R}(-1+\overline{R}^2-\overline{z}^2)(-a\overline{z}W + b(2+\overline{R}^2+2Z^2))(\overline{z}\widehat{\mathbf{R}} + \widehat{\boldsymbol{\phi}})\\ &\quad + \overline{R}(b-a)(a\overline{z}W^2 - b(\overline{R}^2(-3+\overline{z}^2) + 2\overline{z}W^2))(\overline{z}\widehat{\mathbf{R}} + \widehat{\boldsymbol{\phi}})\\ &\quad - \overline{z}W(-b^2\overline{R}^4 + \overline{z}W(a^2+(a-2b)^2\overline{z}^2)\widehat{\mathbf{z}})\Big]. \end{aligned} \qquad \text{(S4.15)}$$

The z-component of this vector, Σ^{b}_z, gives the density covering the Poincaré sphere in a transverse plane. In the focal plane $\overline{z} = 0$, the RH axial C line corresponds to the origin, and around it is the LH C line of radius $\overline{R} = \sqrt{a/b}$, equal to 0.566 with the numerical coefficients. Since the vortex sign of the LH component is negative, the C point at the origin has a 'star' form [15, 16] – α decreases as ϕ increases in a right-handed sense around the beam axis.

With the numerical values of the coefficients, the integral of Σ_z^{b} in the focal plane, over the disk to radius $\sqrt{a/b}$ gives -1, equivalent to covering the Poincaré sphere once in a negative sense. This is consistent with the azimuthal sense of $\nabla_\perp\alpha$ being against $\widehat{\boldsymbol{\phi}}$ while $\nabla_\perp(\cos\phi) = \nabla_\perp S_3$ points towards the origin within this disk, ensuring $\widehat{\mathbf{z}}\cdot\nabla_\perp\alpha\times\nabla_\perp(\cos\beta) < 0$. Outside the radius of the C line loop, the sign of $\nabla_\perp S_3$ changes although $\nabla_\perp\alpha$ does not, so $\Sigma_z^{\mathrm{b}} > 0$; since, asymptotically as $\overline{R} \gg 1$, the polarization state is RH, the net baby Skyrme number (degree of the map covering the Poincaré sphere) is zero.

The magnitude of the baby Skyrme density in the focal plane near the circular C line is large; within the circles where $S_3 = \cos\beta = 0$, at $\overline{R} = 0.524, 0.610$ with the numerical coefficients, corresponds to covering the entire lower hemisphere, whereas the entire upper hemisphere is covered inside and outside this annulus.

By topological continuity, the baby Skyrme number in every other transverse plane is also zero (the radial asymptotic state of polarization being RH circular ensures this is always well-defined). As the LH circular polarization is restricted only to the C line loop in the focal plane, the baby Skyrme number must be zero in every transverse plane except the focal plane.

The 3D Skyrme number involves the product of Σ_z^{b} with $\nabla\gamma$. Since the C line is a phase vortex in γ, $\nabla\gamma$ changes sign on each side of the circular C line, implying the 3D Skyrme density Σ maintains the same sign although Σ_z^{b} changes sign.

We briefly remark on the comparison between the 2D baby skyrmions in our transverse beam profiles and the 'Néel type' (radial) and 'Bloch type' (spiral) baby skyrmions studied in magnetic systems [34]. In a magnetic system, (baby) skyrmions are localised, nonlinear excitations of a ground state (corresponding, e.g. to LH polarization) with Skyrme number 1 in our terminology; the 'type' indicates whether azimuth angles correspond to ϕ with an offset or not. In our beams, in the focal plane, $\phi = -\alpha$, so there is no spiral-like contribution: the baby skyrmion configuration in the focal plane is purely radial (Néel). However, in other planes, α acquires an offset (which is dependent on radius) due to the phase difference between E_{R} and E_{L}, meaning in a typical plane it appears Bloch-type. Of course, for polarization, the distinction between radial and spiral is relative; since polarization is a pseudospinor, the relation between polarization azimuth α and real space azimimuth ϕ is a matter of convention.

4.4.3. Polarization hopfion

We now consider our model field purely as a hopfion, i.e. how the Poincaré sphere is covered by the polarization states in the focal volume. As a map from 3D (real space) to the Poincaré 2-sphere, the preimage of each polarization state is a 1D line – these are the polarization filaments discussed in the main text, generalising C lines for circular polarization.

For an ideal hopfion, the filaments should correspond to a Hopf fibration in real space: loops wound around tori on which $S_3 = \cos\beta$ is constant; these tori enclose the LH circular C line loop. The contours of S_3 from equation (S4.13) in a plane of fixed azimuth is shown in Supplementary Fig. 2c. Evidently, $S_3 = -1$ on the LH C line loop (reference circle) with $\overline{R} = r_0 = \sqrt{a/b}$. The ideal hopfion structure would have the contours of constant S_3 loops around this point, topologically equivalent to the contours of $|u|$ in Supplementary Fig.2a. With the numerical choice of a and b as discussed in Supplementary Note 2, S_3 has saddle points at $(\overline{R}, \overline{z}) = (2\sqrt{b}, \pm\sqrt{a+b})/\sqrt{3b-a} = (1.22, \pm 0.702)$, at $S_3 = 1 - 8c^2/((a-b)^2 + 4c^2) = 0.969$.

At values of S_3 larger than this value, $0.969 < S_3 \leq 1$, the toroidal structure beaks down, into topological cylinders extending to $\overline{z} \to \pm\infty$; topologically the hopfion structure breaks down here, although this counts for only 1.5% of the range of $S_3 = \cos\beta$.

As discussed above, the Hopf number of the hopfion can only be calculated with the choice of a suitable phase function $\widetilde{\gamma}$; using the physical phase γ gives the skyrmionic hopfion, but the global integral of $\nabla\widetilde{\gamma}\cdot\Sigma^{\mathrm{b}}$ should not change under a gauge transformation $\widetilde{\gamma} \to \widetilde{\gamma}'$. As noted above, the physical phase fails to converge due to the LG beams' wavefront curvature, so we will first consider the physical phase gradient in more detail.

4.4.4. Physical phase gradient

The phase parameter γ is the sum of phases of the two beam components, from equation (S1.13). Its gradient therefore is a sum of phase gradients from different phase features of the full field, as superposition of LG beams,

$$\nabla\gamma \;=\; \mathbf{g}^{\mathrm{r}} + \mathbf{g}^{\mathrm{a}} + \mathbf{g}^{\mathrm{G}} + \mathbf{g}^{\mathrm{c}}, \tag{S4.16}$$

where $\mathbf{g}^{\mathrm{r}}$ is associated with the phase of the C line ring, $\mathbf{g}^{\mathrm{a}}$ with the axial C line, $\mathbf{g}^{\mathrm{G}}$ with the Gouy phase, and $\mathbf{g}^{\mathrm{c}}$ with the wavefront curvature. The separation into these terms is very natural for our model, consisting of a superposition of three LG beams, with $(\ell, p) = (0,0), (0,1)$ and $(-1,0)$. All of these have the same wavefront curvature factor, and the axial phase is due to the negative vortex in the LH component. Since the beam with $(\ell, p) = (0,1)$ dominates the

superposition for large R and z, we choose the Gouy phase of this beam to represent $\mathbf{g}^{\mathrm{G}}$; the phase for the ring $\mathbf{g}^{\mathrm{r}}$ takes all the other phase contributions, including the contributions from the Laguerre polynomials. Only $\mathbf{g}^{\mathrm{r}}$ involves the coefficients of the superposition.

The full Skyrme density $\Sigma = \nabla\gamma\cdot\Sigma^{\mathrm{b}}$ can therefore be considered as the contribution of separate terms, $\Sigma^{\bullet} = \mathbf{g}^{\bullet}\cdot\Sigma^{\mathrm{b}}$, for $\bullet = \mathrm{r, a, G, c}$ respectively. The important contributions for the skyrmion topology are those phases corresponding to phase singularities in the physical field (and singularities in α), that is, from the C lines. These are therefore $\mathbf{g}^{\mathrm{r}}$ and $\mathbf{g}^{\mathrm{a}}$.

The various phase gradients are readily found to be

$$\left.\begin{array}{rcl}\mathbf{g}^{\mathrm{r}} &=& \frac{2(a-b)}{((a-b\overline{R}^2)^2+2(a^2-ab(2-\overline{R}^2)+2b^2(1-R^2))\overline{z}^2+(a-2b)^2\overline{z}^4)}\left(2bR\overline{z}\widehat{\mathbf{R}}+(a-bR^2+(a-2b)\overline{z}^2)\widehat{\mathbf{z}}\right),\\ \mathbf{g}^{\mathrm{a}} &=& -\widehat{\boldsymbol{\phi}}/\overline{R},\\ \mathbf{g}^{\mathrm{G}} &=& -5W\widehat{\mathbf{z}}\\ \mathbf{g}^{\mathrm{c}} &=& 2\overline{R}\overline{z}\widehat{\mathbf{R}}/W+R^2(1-\overline{z}^2)\widehat{\mathbf{z}}/W^2.\end{array}\right\} \quad \text{(S4.17)}$$

Evidently, from equation (S4.12), $\nabla\alpha = \mathbf{g}^{\mathrm{a}} - \mathbf{g}^{\mathrm{r}} + (1+\overline{z}^2)^{-1}\widehat{\mathbf{z}}$; the final term accounts for the difference in Gouy phases of the RH and LH beams, and otherwise $\nabla\alpha$ is the difference of the axial and ring phase gradients whilst $\nabla\gamma$ includes their sum, just like how, as described in Supplementary Note 1, $\arg U$ and $\arg V$ are the sum and difference of α and γ for the Hopf fibration of the optical hypersphere.

Both Σ^{r} and Σ^{a} must therefore be included in any function determining the Hopf density of the full field. Numerical integration of the cut volumes of $|\overline{x}|, |\overline{y}| \leq 1.84$, $|\overline{z}| \leq 0.768$ gives 0.4964 and 0.4967 for the axial and ring contributions to the Skyrme number, adding to 0.9931.

The other phase gradients $\mathbf{g}^{\mathrm{G}}$ and $\mathbf{g}^{\mathrm{c}}$ arise from the physics of propagating gaussian beams. The Gouy phase $\mathbf{g}^{\mathrm{G}}$ is purely in the z-direction, so the corresponding Hopf density Σ^{G} is proportional to the transverse baby Skyrme density (as indeed would be any phase associated with the $\mathrm{e}^{\mathrm{i}kz}$ propagation); as discussed above, its integral over any transverse plane is zero, meaning the integral of Σ^{G} over all space is zero. Within the investigated volume, $\int_{\mathrm{vol}}\Sigma^{\mathrm{G}}\,\mathrm{d}^3\mathbf{r} = 0.0014$, making an almost negligible contribution.

Unlike the other contributions to $\nabla\gamma$, the wavefront curvature phase gradient $\mathbf{g}^{\mathrm{c}}$ diverges as $\overline{R} \gg 1$, since the phase curvature does not settle to a constant value for $\overline{R} \gg 1$ when $\overline{z} \neq 0$. In this regime, $\Sigma^{\mathrm{c}} \sim c^2/\pi^2 b^2\overline{R}^2$, accounting for the total logarithmic divergence in the total Σ. This contribution accounts for why the integrated Skyrme density exceeds 1 for much larger volumes, as can be seen in Supplementary Fig. 8b when the boundaries $L_\perp$ and $L_\parallel$ are quite large.

Although mathematically a divergence, the actual divergence is very slow, becoming large where the beam intensity is low, and thus hard to measure physically. In fact, the paraxial approximation of the physical beam in terms of the mathematical model breaks down for sufficiently large $\overline{R}$ and $\overline{z}$. Within the cut volume as described above, the wavefront curvature contributes 0.0020 to the Skyrme number; slightly larger than the Gouy phase, but still very small.

The sum of these four contributions gives the value of the Skyrme number numerically integrated over the cut volume, as described above: 0.4964+0.4967+ 0.0015+0.0020 = 0.9966.

4.4.5. Global Hopf number

From the previous section, it is natural to calculate the global Hopf number as the integral over all space of $\Sigma^{\mathrm{a}} + \Sigma^{\mathrm{r}}$; these include the important topological helicity terms and do not diverge at infinity. With the numerical values of the coefficients, this global Hopf number in fact gives zero; the nontrivial degree of the localised hopfion within the measured volume is, mathematically, unwound within the larger volumes, beyond the saddle of $\cos\beta$ in $\overline{R}, \overline{z}$.

This does not remove the "particle-like" nature of the measured hopfion, although the precise numerical value depends on precisely which phase contributions are included; unlike the Skyrme density, which is physically defined in any volume, the more general Hopf density is only unambiguous within the infinite volume.

Preliminary numerical investigations of beams with different values of a, b, c, not constrained by being realised in experiment, indicate that configurations with nonzero global Hopf number are possible. This behavior of the global Hopf number indicates some subtlety in the global topology of such paraxial LG beam superpositions which would merit further investigation.

4.4.6. Ideal skyrmion field and higher Skyrme numbers

We conclude this section with a short consideration of the ideal skyrmion based on u and v defined in equation (S2.7),

$$\left.\begin{array}{rcl} E_{\mathrm{R}} &=& \left(\frac{R^2+z^2-1+2\mathrm{i}z}{R^2+z^2+1}\right)^n = \operatorname{sech}^n\tau\,\mathrm{e}^{\mathrm{i}n\sigma},\\ E_{\mathrm{L}} &=& \left(\frac{2R\,\mathrm{e}^{\mathrm{i}\phi}}{R^2+z^2+1}\right)^m = \tanh^m\tau\,\mathrm{e}^{\mathrm{i}m\phi},\end{array}\right\} \quad \text{(S4.18)}$$

where m, n are nonnegative integers, the first equalities define the fields in terms of cylindrical coordinates R, ϕ, z, and the second equalities in terms of toroidal coordinates [11–13], similar to Supplementary Note 1 but here applied to real 3D space. We perform the calculation here in toroidal coordinates, which is very natural for the ideal skyrmion field. This can be compared to the similar calculation in [55], which assumes spherical symmetry.

The parameters of this definition of the field, from equations (S1.10), (S1.11) and (S1.13) are easily expressed in toroidal coordinates,

$$\gamma = m\phi + n\sigma, \qquad \alpha = m\phi - n\sigma, \qquad \cos\beta = \frac{\mathrm{sech}^{2n}\tau - \tanh^{2n}\tau}{\mathrm{sech}^{2n}\tau + \tanh^{2n}\tau}. \tag{S4.19}$$

Continuing in toroidal coordinates, the gradients are

$$\left.\begin{aligned} \nabla\gamma &= (\cosh\tau - \cos\sigma)(m\hat{\boldsymbol{\phi}}/\sinh\tau + n\hat{\boldsymbol{\sigma}}), \\ \nabla\alpha &= (\cosh\tau - \cos\sigma)(m\hat{\boldsymbol{\phi}}/\sinh\tau - n\hat{\boldsymbol{\sigma}}), \\ \nabla(\cos\beta) &= -4(\cosh\tau - \cos\sigma)\,\mathrm{csch}\,\tau\,\mathrm{sech}^{2m+1}\tau\tanh^{2n}\tau\,\hat{\boldsymbol{\tau}}/(\mathrm{sech}^{2n}\tau + \tanh^{2n}\tau)^2. \end{aligned}\right\} \tag{S4.20}$$

The volume element for toroidal coordinates is $\sinh\tau\,\mathrm{d}\phi\,\mathrm{d}\sigma\,\mathrm{d}\tau/(\cosh\tau - \cos\sigma)^3$, so the Skyrme density $\Sigma = \nabla\gamma\cdot\nabla\alpha\times\nabla(\cos\beta)$, and dividing by the 3-sphere volume $16\pi^2$, gives the total integrated Skyrme number

$$\int_0^\infty\int_{-\pi}^{\pi}\int_{-\pi}^{\pi}\frac{\Sigma\sinh\tau}{16\pi^2(\cosh\tau-\cos\sigma)^3}\,\mathrm{d}\phi\,\mathrm{d}\sigma\,\mathrm{d}\tau = mn\int_0^\infty\frac{\mathrm{d}}{\mathrm{d}\tau}\left[\frac{1}{1+\cosh^{2n}\tau\tanh^{2m}\tau}\right]\mathrm{d}\tau = -mn, \tag{S4.21}$$

where, since the net integrand is dependent only on τ, the angle integrals are trivial. The net integral is identified as a pure derivative, so the overall τ integral is also straightforward, leaving a net result mn from the phase windings in equation (S4.18). Choosing the conjugate of either of the fields in the definition (S4.18) gives a positive Skyrme density, as in previous discussion where $m = n = 1$ and a negative axial vortex was chosen.

This is instructive for designing optical skyrmionic hopfions of higher Skyrme number, e.g. using superpositions of LG beams. The strategy is similar to that employed by [56], who designed scalar fields with vortex loops threaded by vortices on the axis. Increasing the axial degree m is easy, by replacing the LH component with a different azimuthal quantum number $\ell = m$. However, as described in [56], paraxial beams do not allow transverse vortex loops with $|n| > 1$, Realising a higher-order skyrmion with effective $|n| > 1$ is possible with multiple vortex loops with the same sense of vorticity, and indeed, these can be woven into various kinds of link and knot as constructed in [47, 48].

Supplementary Note 5: Details of numerical methods

Given the the map from real space to the optical hypersphere described in the numerical calculation of the experimental Skyrme Number (see Methods), we can visualise the optical skyrmionic hopfion and its Skyrme density. We imagine each measured point in real space to correspond to the vertices of a cubic lattice, which is carried to an irregular lattice with the same topology in the optical hypersphere. In regions of the beam where the Skyrme density is high, such as close to the circular C line, the corresponding spherical tetrahedra (and cubes comprised of them) are large, and where the Skyrme density is small so are the spherical tetrahedra. Explicative examples are the grey cubes in Fig. 4 of the main text: in real space (Fig. 4a) the cubes have the same size, whereas in the optical hypersphere, (Fig. 4b), the cube enclosing the C line loop (in black) is much larger than the other cube. Supplementary Fig. 9, a and b, show the analogous mapping for the ideal skyrmion field. Main text Fig. 4c shows all of the sampled cubes in real space, whose colour represents Vol(C): the transparency goes to 0 for very small volumes, becomes increasingly orange for positive volumes and increasingly blue for negative volumes. Our algorithm is based on expressing the measured data as the vertices of tetrahedral meshes (i.e. 3D cell complexes) in real space and the optical hypersphere. The calculations of the spherical tetrahedra volumes in the 3-sphere are described, with a final section describing how this is found from the image of the boundary of the measured volume.

5.1. The volume of a spherical tetrahedron

The 3-sphere is embedded in four-dimensional Euclidean space. Four unit vectors, $\vec{n}_\ell$ with $\ell = \{a, b, c, d\}$, point to the four vertices of a spherical tetrahedron T. The four faces of this spherical tetrahedron are spherical triangles, pairs of

which meet along edges at the dihedral angles $0 < \varphi_j < \pi$ for $j = 1, 2, \dots, 6$. The formula for $\mathrm{Vol}(T)$ [57], 3D volume of the tetrahedron in the hypersphere, is in terms of the dihedral angles.

Given $a_j = \exp(\mathrm{i}\varphi_j)$ and the dilogarithm function $\mathrm{Li}_2(z)$, the volume of the spherical tetrahedron is, from Murakami's formula [57],

$$\mathrm{Vol}(T) = -\,\mathrm{Re}(\mathrm{L}(a_1, a_2, a_3, a_4, a_5, a_6, z_0)) + \pi \left(\arg(-q_2) + \frac{1}{2}\sum_{j=1}^{6} \varphi_j \right) - \frac{3}{2}\pi^2 \bmod 2\pi^2, \quad \text{(S5.1)}$$

where

$$\begin{aligned} \mathrm{L}(a_1, a_2, a_3, a_4, a_5, a_6, z) = \frac{1}{2} \Big(& \mathrm{Li}_2(z) + \mathrm{Li}_2(a_1^{-1}a_2^{-1}a_4^{-1}a_5^{-1}z) + \mathrm{Li}_2(a_1^{-1}a_3^{-1}a_4^{-1}a_6^{-1}z) + \mathrm{Li}_2(a_2^{-1}a_3^{-1}a_5^{-1}a_6^{-1}z) \\ & -\mathrm{Li}_2(-a_1^{-1}a_2^{-1}a_3^{-1}z) - \mathrm{Li}_2(-a_1^{-1}a_5^{-1}a_6^{-1}z) - \mathrm{Li}_2(-a_2^{-1}a_4^{-1}a_6^{-1}z) - \mathrm{Li}_2(-a_3^{-1}a_4^{-1}a_5^{-1}z) + \sum_{j=1}^{3} \log a_j \log a_{j+3} \Big) \quad \text{(S5.2)} \end{aligned}$$

and

$$\begin{aligned} z_0 &= -q_1 + \sqrt{q_1^2 - 4q_0q_2}/(2q_2), \\ q_0 &= a_1a_4 + a_2a_5 + a_3a_6 + a_1a_2a_6 + a_1a_3a_5 + a_2a_3a_4 + a_4a_5a_6 + a_1a_2a_3a_4a_5a_6, \\ q_1 &= -(a_1 - a_1^{-1})(a_4 - a_4^{-1}) - (a_2 - a_2^{-1})(a_5 - a_5^{-1}) - (a_3 - a_3^{-1})(a_6 - a_6^{-1}), \\ q_2 &= a_1^{-1}a_4^{-1} + a_2^{-1}a_5^{-1} + a_3^{-1}a_6^{-1} + a_1^{-1}a_2^{-1}a_6^{-1} + a_1^{-1}a_3^{-1}a_5^{-1} + a_2^{-1}a_3^{-1}a_4^{-1} + a_4^{-1}a_5^{-1}a_6^{-1} + a_1^{-1}a_2^{-1}a_3^{-1}a_4^{-1}a_5^{-1}a_6^{-1}. \end{aligned} \quad \text{(S5.3)}$$

The dihedral angles are found from the tetrahedron's vertices using hyperspherical trigonometry as follows [58]. For each triple of hyperplanes with normals $\vec{n}_i, \vec{n}_j, \vec{n}_k$, define the vector $\vec{u}_{ijk}$ orthogonal to each hyperplane, by

$$\vec{u}_{ijk} = (\vec{n}_i \times \vec{n}_j \times \vec{n}_k)/|\vec{n}_i \times \vec{n}_j \times \vec{n}_k|.$$

Here, the ternary vector product in four dimensions, $(\vec{a} \times \vec{b} \times \vec{c})$, can be defined as $(\vec{a} \times \vec{b} \times \vec{c}) \cdot \vec{e}_i = \det(\vec{a}, \vec{b}, \vec{c}, \vec{e}_i) = \varepsilon_{ijkl}a_jb_kc_l$, where $\vec{e}_i$ are the unit vectors in four dimensions and ε_{ijkl} is the Levi-Civita symbol [58]. This is related to the 4D volume form considered in Supplementary Note 1.

Each dihedral angle φ_{jk} satisfies $\cos\varphi_{jk} = \vec{u}_{ijk} \cdot \vec{u}_{jkl}$. The two indices of the dihedral angles are simplified to a unique index $j = (1, 2, \dots, 6) = (da, db, cd, bc, ac, cb)$, which, when substituted into the formula (S5.1), gives the volume of a single tetrahedron.

5.2. Visualising the covered volume of optical hypersphere

We previously showed in Supplementary Note 4 that the integrated Skyrme density for the same measured volume corresponds to is 0.997 of the hypersphere volume. This difference can be accounted for from experimental imperfections and the Skyrme number. Topologically, the difference must be related to the images of the measured volume boundary in the hypersphere.

The optical states on the boundary of the measured volume are to some extent arbitrary. The optical state $(E_\mathrm{R}, E_\mathrm{L}) = (-1, 0)$ (i.e. $\omega = \pi$) occurs at the focal point, and as $R \to \infty$ in the focal plane $z = 0$, $(E_\mathrm{R}, E_\mathrm{L}) = (+1, 0)$ (i.e. $\omega = 0$). The $\mathrm{LG}_{0,1}$ contribution to E_R dominates the field for large $R \gg w$, so for an appropriately large measurement volume (although not too large, for reasons discussed in Sec. 4.4), the boundary of the measurement volume consists of optical states close, in the optical hypersphere, to the state $(1, 0)$, which is antipodal to the state at the focal point.

The image of the measurement boundary separates the optical hypersphere into two disjoint volumes: that covered by the measured optical states (covering 94 % experimentally), and a neighbourhood of the state $(1, 0)$, covering approximately 6 % of the hypersphere. We chose to represent the optical hypersphere, in main text Figs. 3 and 4, using the volume-preserving projection with the optical state at the focal point, $(-1, 0)$. The antipodal state $(-1, 0)$ therefore corresponds to the sphere boundary of the solid ball. The image of the measured boundary, being a neighbourhood of this point, therefore would appear to be a sphere close to the boundary sphere in this projection. The boundary of the

measured volume, that is, the image of the six faces of the measured cubic volume, are plotted in the described projection of the optical hypersphere in Supplementary Fig. 10a. Clearly this is a surface that almost coincides with the boundary of the projection at $(1, 0)$. The corresponding plot for the theoretical model field is shown in the inset.

In this projection it is hard to see that the volume of this neighbourhood complementing the filled volume is small. For this reason, in Supplementary Fig. 10b, we plot the optical hypersphere in a different projection, with a point on the circular C line at the center (and the opposite point on the C line loop to the boundary of the ball). The RH states on the axis in real space become a circle in this projection, and it is clear here that the volume of the optical hypersphere not covered is small (consistent with the theoretical model, inset to Supplementary Fig. 10b).

Supplementary References

[1] Born, M. and Wolf, E. *Principles of Optics*. Cambridge University Press, 7th edition, (1999).
[2] Mansuripur, M. *Classical Optics and its Applications*. Cambridge University Press, (2009).
[3] Dennis, M. R. *Topological Singularities in Wave Fields*. PhD dissertation, University of Bristol, (2001).
[4] Dennis, M. R. Polarization singularities in paraxial vector fields: morphology and statistics. *Opt. Commun.* **213**, 201–221 (2002).
[5] Goldstein, H. *Classical Mechanics*. Pearson, 2nd edition edition, (1980).
[6] Hannay, J. H. Cyclic rotations, contractibility and Gauss-Bonnet. *J. Phys. A: Math. Gen.* **31**, L321–L324 (1998).
[7] Weeks, J. R. *The Shape of Space*. Marcel Dekker, New York, (1985).
[8] Henderson, D. W. and Taimina, D. *Experiencing Geometry: in Euclidean, Spherical, and Hyperbolic Spaces*. Prentice Hall, (2001).
[9] Frank, F. C. Orientation mapping. *Metall. Mater. Trans. A* **19**, 403–408 (1988).
[10] Frank, F. C. The conformal neo-eulerian orientation map. *Philos. Mag. A* **65**, 1141–1149 (1992).
[11] Byerly, W. E. *An elementary treatise on Fourier's series and spherical, cylindrical, and ellipsoidal harmonics: with applications to problems in mathematical physics*. Ginn & Co., Boston, (1895).
[12] Bateman, H. *Partial Differential Equations of Mathematical Physics*. Dover, (1944).
[13] Moon, P. and Spencer, D. E. *Field Theory Handbook*. Springer-Verlag Berlin Heidelberg, (1988).
[14] Siegman, A. E. *Lasers*. University Science Books, (1986).
[15] Nye, J. F. *Natural Focusing and Fine Structure of Light: Caustics and Wave Dislocations*. IoP Publishing, (1999).
[16] Dennis, M. R., O'Holleran, K., and Padgett, M. J. Singular Optics: Optical Vortices and Polarization Singularities. *Prog. Optics* **53**, 293–363 (2009).
[17] Dennis, M. R., King, R. P., Jack, B., O'Holleran, K., and Padgett, M. J. Isolated optical vortex knots. *Nat. Phys.* **6**, 118–121 (2010).
[18] Dennis, M. R., Goette, J. B., King, R. P., Morgan, M. A., and Alonso, M. A. Paraxial and nonparaxial polynomial beams and the analytic approach to propagation. *Opt. Lett.* **36**, 4452–4454 (2011).
[19] Bauer, T., Banzer, P., Karimi, E., Orlov, S., Rubano, A., Marrucci, L., Santamato, E., Boyd, R. W., and Leuchs, G. Observation of optical polarization Möbius strips. *Science* **347**, 964–966 (2015).
[20] Larocque, H., Sugic, D., Mortimer, D., Taylor, A. J., Fickler, R., Boyd, R. W., Dennis, M. R., and Karimi, E. Reconstructing the topology of optical polarization knots. *Nat. Phys.* **14**, 1079–1082 (2018).
[21] Sugic, D. *Unravelling the dark focus of light: a study of knotted optical singularities*. PhD dissertation, University of Bristol, (2019).
[22] Otte, E., Rosales-Guzmán, C., Ndagano, B., Denz, C., and Forbes, A. Entanglement beating in free space through spin-orbit coupling. *Light: Sci. & Appl.* **7**, 18007–18009 (2018).
[23] Schulze, C., Flamm, D., Duparré, M., and Forbes, A. Beam-quality measurements using a spatial light modulator. *Opt. Lett.* **37**, 4687–4689 (2012).
[24] Schaefer, B., Collett, E., Smyth, R., Barrett, D., and Fraher, B. Measuring the Stokes polarization parameters. *Am. J. Phys.* **75**, 163–168 (2007).
[25] Otte, E., Alpmann, C., and Denz, C. Higher-order polarization singularitites in tailored vector beams. *J. Opt.* **18**, 074012 (2016).
[26] Alpmann, C., Schlickriede, C., Otte, E., and Denz, C. Dynamic modulation of Poincaré beams. *Sci. Rep.* **7**, 8076 (2017).
[27] Takeda, M., Ina, H., and Kobayashi, S. Fourier-transform method of fringe-pattern analysis for computer-based topography and interferometry. *J. Opt. Soc. Am.* **72**, 156–160 (1982).
[28] Čižmár, T., Mazilu, M., and Dholakia, K. In situ wavefront correction and its application to micromanipulation. *Nat. Photonics* **4**, 388–394 (2010).
[29] Beckley, A. M., Brown, T. G., and Alonso, M. A. Full Poincaré beams. *Opt. Express* **18**, 10777–10785 (2010).
[30] Donati, S., Dominici, L., Dagvadorj, G., Ballarini, D., De Giorgi, M., Bramati, A., Gigli, G., Rubo, Y. G., Szymańska, M. H.,

and Sanvitto, D. Twist of generalized skyrmions and spin vortices in a polariton superfluid. *Proc. Natl. Acad. Sci. U.S.A.* **113**, 14926–14931 (2016).

[31] Gao, S., Speirits, F. C., Castellucci, F., Franke-Arnold, S., Barnett, S. M., and Götte, J. B. Paraxial skyrmionic beams. *Phys. Rev. A* **102**, 053513 Nov (2020).

[32] Salomaa, M. and Volovik, G. E. Quantized vortices in superfluid He-3. *Rev. Mod. Phys.* **59**, 533–613 (1987).

[33] Leanhardt, A. E., Shin, Y., Kielpinski, D., Pritchard, D. E., and Ketterle, W. Coreless Vortex Formation in a Spinor Bose-Einstein Condensate. *Phys. Rev. Lett.* **90**, 140403 (2003).

[34] Nagaosa, N. and Tokura, Y. Topological properties and dynamics of magnetic skyrmions. *Nat. Nanotechnol.* **8**, 899–911 (2013).

[35] Tsesses, S., Ostrovsky, E., Cohen, K., Gjonaj, B., Lindner, N. H., and Bartal, G. Optical skyrmion lattice in evanescent electromagnetic fields. *Science* **361**, 993–996 (2018).

[36] Du, L., Yang, A., Zayats, A. V., and Yuan, X. Deep-subwavelength features of photonic skyrmions in a confined electromagnetic field with orbital angular momentum. *Nat. Phys.* **15**, 650–654 (2019).

[37] Mermin, N. D. and Ho, T.-L. Circulation and angular momentum in the *A*-phase of superfluid Helium-3. *Phys. Rev. Lett.* **36**, 594–597 (1976).

[38] Bott, R. and Tu, L. W. *Differential Forms in Algebraic Topology*. Springer-Verlag, (1982).

[39] Hatcher, A. *Algebraic Topology*. Cambridge University Press, (2002).

[40] Lee, W., Gheorghe, A. H., Tiurev, K., Ollikainen, T., Möttönen, M., and Hall, D. S. Synthetic electromagnetic knot in a three-dimensional skyrmion. *Sci. Adv.* **4**, eaao3820 (2018).

[41] Shankar, R. Applications of topology to the study of ordered systems. *J. Phys. (France)* **38**, 1405–1412 (1977).

[42] Nakahara, M. *Geometry, Topology and Physics*. Taylor & Francis, 2nd edition edition, (2003).

[43] Manton, N. and Sutcliffe, P. *Topological Solitons*. Cambridge University Press, (2004).

[44] Whitehead, J. H. C. An Expression of Hopf's Invariant as an Integral. *Proc. Natl. Acad. Sci. U.S.A.* **33**, 117–123 (1947).

[45] Moffatt, H. K. and Ricca, R. L. Helicity and the Călugăreanu invariant. *Proc. R. Soc. Lond. A* **439**, 411–429 (1992).

[46] Hietarinta, J., Jaykka, J., and Salo, P. Dynamics of vortices and knots in Faddeev's model. *Proceedings of Workshop on Integrable Theories, Solitons and Duality — PoS(unesp2002)* **008**, 017 (2002).

[47] Kedia, H., Foster, D., Dennis, M. R., and Irvine, W. T. M. Weaving Knotted Vector Fields with Tunable Helicity. *Phys. Rev. Lett.* **117**, 274501 (2016).

[48] Sutcliffe, P. Knots in the Skyrme-Faddeev model. *Proc. R. Soc. A* **463**, 3001–3020 (2007).

[49] Berry, M. V. Optical currents. *J. Opt. A: Pure Appl. Opt.* **11**, 094001 (2009).

[50] Dennis, M. R. On the Burgers vector of a wave dislocation. *J. Opt. A* **11**, 094002 (2009).

[51] Volovik, G. E. and Mineev, V. P. Particle-like solitons in superfluid ^{3}He phases. *JETP Letters* **46**, 401–404 (1977).

[52] Moffatt, H. K. The degree of knottedness of tangled vortex lines. *J. Fluid Mech.* **35**, 117–129 (1969).

[53] Moffatt, H. K. Helicity and singular structures in fluid dynamics. *Proc. Natl. Acad. Sci. U.S.A.* **111**, 3663–3670 (2014).

[54] Winfree, A. T. and Strogatz, S. H. Singular filaments organize chemical waves in three dimensions III. Knotted waves. *Physica D* **9**, 333–345 (1983).

[55] Ruostekoski, J. Stable particlelike solitons with multiply quantized vortex lines in Bose-Einstein condensates. *Phys. Rev. A* **70**, 041601 (2004).

[56] Berry, M. V. and Dennis, M. R. Knotted and linked phase singularities in monochromatic waves. *Proc. R. Soc. A* **457**, 2251–2263 (2001).

[57] Murakami, J. Volume formulas for a spherical tetrahedron. *Proc. Amer. Math. Soc.* **140**, 3289–3295 (2012).

[58] Jennings, P. R. *Hyperspherical Trigonometry, Related Elliptic Functions and Integrable Systems*. PhD dissertation, The University of Leeds, (2013).
