# Supplementary material for: Particle-like topologies in light
Source: Nat Commun. 2021 Nov 22;12:6785. doi: 10.1038/s41467-021-26171-5 (PMC8608860; doi:10.1038/s41467-021-26171-5)
Supplement: Supplementary file 3 — Description of Additional Supplementary Files [file 41467_2021_26171_MOESM3_ESM.pdf]

Description of Additional Supplementary Files

File name: Supplementary Movie 1
Description: Measured skyrmionic hopfion in the focal volume. The measured polarization states are coloured following the Poincaré sphere (see Fig. 1). The video complements Fig. 2 and shows the 3D view of the topological structure of the Hopf fibration, where each filament links once with every other filament. Points corresponding to the same ellipticity ([illegible] = −0.775, 0, 0.398) make up toroidal shells arranged between the two C lines (RH in white and LH in black).
